# Transcript-wide identification and expression pattern analysis to comprehend the roles of AP2/ERF genes under development and abiotic stress in *Trichosanthes kirilowii*

**DOI:** 10.1186/s12870-023-04362-0

**Published:** 2023-07-10

**Authors:** Rui Xiong, Zhuannan Chu, Xingxing Peng, Guangsheng Cui, Weiwen Li, Ling Dong

**Affiliations:** 1 Institute of Horticulture, Anhui Academy of Agricultural Sciences, Hefei, 230001 China; 2 Key Laboratory of Horticultural Crop Germplasm innovation and Utilization (Co-construction by Ministry and Province), Institute of Horticulture, Anhui Academy of Agricultural Sciences, Hefei, 230001 China

**Keywords:** *AP2/ERF*, *Trichosanthes kirilowii*, Abiotic stress, Plant hormone, Expression profile

## Abstract

**Background:**

The APETALA 2/ ethylene-responsive element binding factors (*AP2/ERF*), are thought to be associated with plant abiotic stress response, and involved in some plant hormone signaling pathways. *Trichosanthes kirilowii* is an important edible and medicinal crop, so far no research has been conducted on the *TkAP2/ERF* genes.

**Result:**

In this study, a total of 135 TkERFs were identified, these genes were divided into 4 subfamilies and clustered into 13 groups. Moreover, 37 paralogous pairs were identified, with only two having Ka/Ks values greater than 1, proving that most *TkERF* genes underwent purifying selection during evolution. Co-expression networks constructed using transcriptome data at various flowering stages revealed that 50, 64, and 67 AP2/ERF genes correlated with members of the ethylene, gibberellin, and abscisic acid signaling pathways, respectively. When tissue cultured seedlings were treated with ETH, GA3 and ABA, 11, 12 and 17 genes were found to be up-regulated, respectively, suggesting that some members of the *TkERF* gene family may be involved in plant hormone signaling pathways. And under 4 ℃, PEG and NaCl treatment, 15, 20 and 19 genes were up-regulated, respectively, this suggested that these selected genes might be involved in plant abiotic stresses.

**Conclusions:**

Overall, we identified 135 AP2/ERF family members, a comprehensive analysis of AP2/ERF gene expression patterns by RNA-seq and qRT-PCR showed that they played important roles in flower development and abiotic stress. This study provided a theoretical basis for the functional study of *TkAP2/ERF* genes and the genetic improvement of *T. kirilowii*.

**Supplementary Information:**

The online version contains supplementary material available at 10.1186/s12870-023-04362-0.

## Background

The APETALA 2/ ethylene-responsive element binding factor (*AP2/ERF*) is a plant specific gene family, composed of transcription factors (TFs) [1]. Studies showed that AP2/ERF transcription factors are involved in the regulation of plants abiotic stress and hormone response [2]. According to the number and types of gene domains, the AP2/ERF family can be classified into four subfamilies: ethylene-responsive factors (ERF), dehydration-responsive element binding (DREB), APETALA2 (AP2) and related to ABI3/VP (RAV) [3]. All AP2/ERF transcription factors contain at least an AP2 domain, the AP2 subfamily contains two AP2 domains, the RAV subfamily contains a B3 domain in addition to an AP2 domain; the ERF and DREB subfamilies contain only one AP2 domain, and the difference of the two subfamilies was the amino acids at positions 14 and 19 of the conserved domains. In the ERF subfamily, the 14th and 19th amino acids of AP2/ERF motifs are alanine (Ala) and aspartic acid (Asp), respectively, while the DREB subfamily consists of valine (Val) and glutamate (Glu) at the same positions [4]. Furthermore, according to the classification of *Arabidopsis thaliana*, these two subfamilies can be divided into B1-6 and A1-6 clans respectively [5].

As a large transcription factors family, AP2/ERF has various functions, and different subfamilies play slightly different roles in abiotic stress. Genes in ERF, AP2 and RAV subfamilies mainly play roles in responding to freezing, hypoxia and salt stress, whereas genes in DREB subfamily primarily respond to cold, heat, drought and salt stress [2]. For example, in *A. thaliana*, CRF4 and CRF6 belong to the ERF-VI subfamily, and their function were already studied. *AtCRF4* positively regulated freezing tolerance by promoting the expression of CORs, and *AtCRF6* alleviated H_2_O_2_ damage in plants to positively regulate oxidative response [6]. Another study revealed that two genes in the ERF-VII subfamily, *AtRAP2.2* and *AtRAP2.12*, played major roles in the initial activation of anaerobic responses [7]. In *Oryza sativa*, SUB1A, SNORKEL1 and SNORKEL2, which belong to the EF-VI subfamily, were induced to express in flooded tissues due to enhanced ethylene production. SUB1A can block plant elongation and promote carbohydrate catabolism by increasing the accumulation of SLENDER RICE1 (SLR1) and SLENDER RICE1 LIKE1 [8–10]. While, SNORKEL1 and SNORKEL2 triggered node growth by promoting the expression of GA 20-oxidase [11, 12]. The *Halostachys caspica* TOE3 gene, belongs to the AP2 subfamily, its overexpression in Arabidopsis plants improved plant tolerance to low temperature stress compared with the wild-type [13]. The functions of the DREB subfamily genes were also studied in many plants. In *A. thaliana* and *O. sativa*, the CBF genes belonging to DREB subfamily, were proved to play a role in the early response to plant cold stress by inducing the expression of COR gene [14]. And the expression of *AtDREB2A* was induced by dehydration and heat stress [15]. Meanwhile, when the *TaDREB3* gene was transferred to Arabidopsis plants, the transgenic plants showed resistance to heat, drought and salt stress [16].

Except for the regulation of abiotic stress, AP2/ERF gene family was also related to plant hormone signaling pathways, such as ethylene (ETH), abscisic acid (ABA) and gibberellin (GA3). Meanwhile, these hormones can also directly or indirectly affect plant growth and development. In ETH transduction pathway, the expression of ERF transcription factorwas regulated by EIN3 [17]. Interestingly, the ACS gene which was involved in ethylene synthesis pathway, ERF can bind to its promoter and regulate the transcription, thus affectingsexual differentiation in many plants [18, 19]. The *AtERF53*, *AtRAP2.6L* and *AtRAP2.6* can be induced by ABA [20–22]. And OsERF71 was demonstrated to positively modulate ABA signaling pathway [23]. Interactions between ERF and some genes in ABA transduction pathway have been observed. For examples, *AtERF18* can activate the PP2C family phosphatase gene ABI2 [24]; *AtRAV1* can bind to the *ABI3*, *ABI4* and *ABI5* promoters and inhibit their expression; while *SNRK2* (*SNRK2.2*, *SNRK2.3* and *SNRK2.6*), another enzyme in the ABA transduction pathway, can phosphorylate *AtRAV1* [25]. For GA signaling pathway, *AtERF11* was proved to promote the expression of *AtGA3ox1* and *AtGA20oxs*, thereby activating GA biosynthesis and promoting plant internode elongation [26]. While overexpression of *AtERF6* results in extreme dwarf phenotype, and proved that antagonism between *AtERF6* and *AtERF11* [27]. The role of AP2/ERF genes in affecting GA signaling pathway were also demonstrated in other plants, such as *Solanum lycopersicum* [28], *Physic nut* [29], and *O. Sativus* [30].

*Trichosanthes kirilowii* Maxim belonging to the cucurbitaceous, is a perennial climbing dioecious herb. Its roots, pericarp and seeds are all be used in traditional Chinese medicine. In recent years, *T. kirilowii* formulation was increasingly applied in the tumor and cardiovascular diseases clinical treatment. Due to increasing demand, *T. kirilowii* was widely planted in Anhui, Shanxi, Gansu, Sichuan, Guizhou and Yunnan provinces of China. However, most varieties have no resistance to disease, insect pests and drought. ERF, a gene family which is known to be associated with abiotic stress-responsive, plant growth and development, was necessary to be studied for the cultivation of new and improved varieties. *T. kirilowii*, as an octaploid plant, the whole genome has not yet been sequenced, molecular mechanism related to plant growth can only studied by transcriptome sequencing.

In this study, we identified 135 TkAP2/ERF genes through the transcript data. We conducted phylogenetic analyses, conserved motif and divided the TkAP2/ERF genes into four subfamilies with 13 clans. The ERF expression pattens during different flowers periods were analyzed. To screen for potentially functional genes, we mapped the co-expression networks of differentially expressed TkAP2/ERF genes and genes involved in ETH, GA3 and ABA signaling pathways. Finally, we conducted real-time PCR analysis to examine the relative expression of AP2/ERF genes in *T. kirilowii* plants under various abiotic stresses (low temperature, drought, and cold) and hormone treatment (ABA, ETH, GA3). Our results can provide a reference for the study of *TkERF* gene function. 

## Results

### Identification of AP2/ERF genes in *T. kirilowii*

Based on the hidden Markov model (HMM) profiles (PF00847) and homology searches, 135 members of *TkERF* gene family were identified. Information of *T. kirilowii ERF* genes, including open reading frame (ORF) length, protein length, isoelectric point (PI), molecular weight (MW) and subcellular localization were listed in Table S1. The ORF length ranged from 255 bp (*TkERF77*) to 2112 bp (*TkAP2-1*), the protein length ranged from 84 to 703 aa, the predicted molecular weights ranged from 8888.86 Da (TkERF77) to 539953.22 Da (TkAP2-30). The PI values of all *TkERF* genes were ranged from 4.61 (TkAP2-30) to 12 (TkERF29). Subcellular localization analysis of the AP2/ERF gene products was also performed (Table S1). Five genes were located in chloroplast, one gene was located in mitochondria, two genes were located in cytoplasm, and 127 genes were located in nucleus.

### Phylogenetic analysis and classification of TkERF genes

**Fig. 1:**
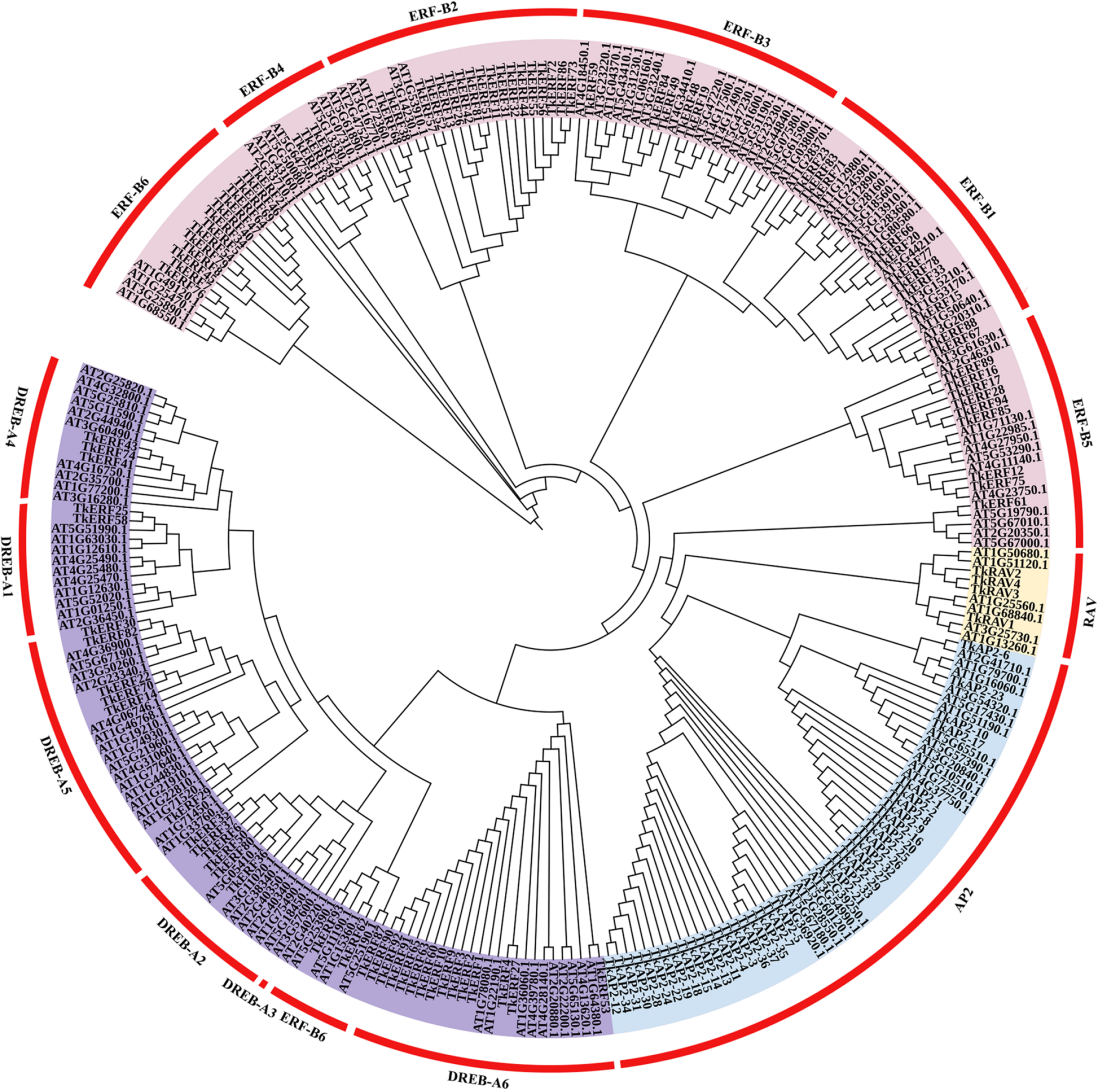
Phylogenetic tree of full-length *AP2/ERF* genes from *Trichosanthes kirilowii* and *Arabidopsis thaliana*. The tree was constructed using the neighbor-joining (NJ) method with MEGA 11.0 based on AP2/ERF sequences from the *Trichosanthes kirilowii* (135 proteins) and the *Arabidopsis thaliana* (145 proteins). The AP2/ERF family members were categorised based on the previous classifications (Toshitsugu et al., 2006; Nakano et al., 2006)and marked by a distinct color

To explore the evolutionary relationships between *A. thaliana* and *T. kirilowii,* an unrooted neighbor-joining (NJ) phylogenetic tree of 135 *TkERF* and 145 *AtERF* genes was constructed (Fig. 1). According to the AtERF classification, TkERF genes were divided into four subfamilies: ERF, DREB, AP2 and RAV. In *T. kirilowii*, ERF subfamily contained 63 members, DREB subfamily contained 31 members, 37 genes which containing two AP2/ERF domains were defined as AP2 subfamily, and RAV which contained one AP2/ERF and one B3 domain were consisted of 4 members.

### Conserved motif and selective pressure analyses of *TkERF* genes

**Fig. 2:**
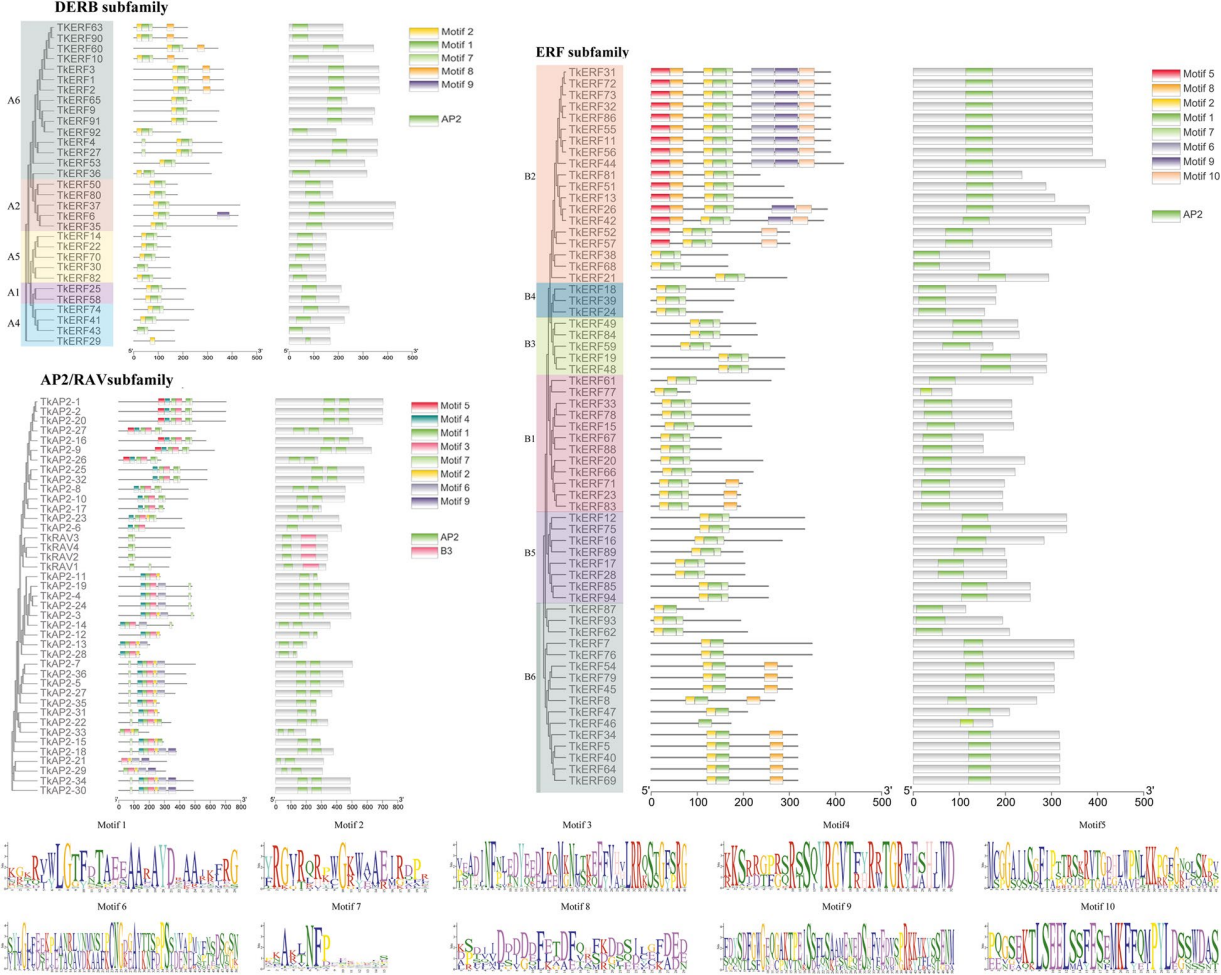
Domain, motif compositions and distribution of conserved motifs TkAP2/ERF proteins. The conserved domains AP2 and B3, which were identified using the CDD online research, were indicated by green and red boxes, respectively. A schematic representation of the conserved groups was illustrated using MEME. The colored box represents the motif. Seq logo plots of 10 identified motifs generated using batch MEME motifs viz program

To investigate the conserved domain characteristics of TkERF/AP2 protein, motif analysis of 135 genes was performed using Multiple Expectation Maximization for Motif Elicitation (MEME) online tools, and 10 motifs were identified (Fig. 2). As shown in Fig. 2, genes in the same subfamily contained essentially same type and order of motif. Motif 1, motif 2 and motif 7 appears in almost all genes except for DREB-A4 Clan and RAV subfamily, and the motif 1 and motif 2 were the AP2 DNA-binding motifs. In ERF subfamily B1 and B6 clans, part of the gene also contains motif 10; in addition to the three motifs mentioned above, most genes in B2 clan also contained motif 5, motif 6, motif 8 and motif 9. The motifs of DREB subfamily were more conservative. Except for the motif 8 in A4, almost all other clans only have motif 1,2 and 7. RAV subfamily members contained only motif 1 and motif 7. The motifs which were contained in AP2 were different from the other three subfamilies. Some of the genes contained motif 1, motif 3, motif 4, motif 5 and motif 7; while others also contained motif 6, five genes contained motif 6 and motif 9.

Homologous pairs of ERF genes were identified between *T. kirilowii* and *A. thaliana*, and were listed in Table 1. Thirty-three paralogous pairs in *T. kirilowii,* 17 orthologous pairs between *T. kirilowii* and *A. thaliana* were identified. To better understand Darwinian evolutionary selection for the TkERF gene family, we calculated non-synonymous substitution (Ka), synonymous substitution rate (Ks), Ka/ Ks values of the pairs (Tables 1 and 2). The results showed that only two paralogous pairs (*TkERF9*/*TkERF92*, *TkERF14*/*TkERF22*) had Ka/Ks values greater than 1, this indicated that a few TkERF genes undergone positive slection. The Ka/Ks ratios of the remaining homologous pairs were all less than 1, suggesting that the *TkERF* gene has undergone strong purification selection.

**Table 1:** Ka, Ks and Ka/Ks values for the AP2/ERF genes in Trichosanthes

Paralogous pairs	Ka	Ks	Ka/Ks
TkERF1/ TkERF60	0	0.011976	0
TkERF2/ TkERF60	0.00653	0.040938	0.159517
TkERF4/ TkERF27	0.006168	0.019941	0.309329
TkERF7/ TkERF76	0.004885	0.023141	0.211076
TkERF8/TkERF45	0.087128	0.143827	0.605781
TkERF9/ TkERF92	0.077307	0.073641	1.049774
TkERF12/ TkERF75	0.005274	0.013053	0.404005
TkERF14/ TkERF22	0.023889	0.019529	1.223224
TkERF15/ TkERF78	0.202012	1.387938	0.145548
TkERF15/ TkERF33	0.205087	1.299267	0.157848
TkERF16/TkERF89	0.047165	0.068679	0.686737
TkERF17/TkERF94	0.008825	0.034789	0.253681
TkERF17/TkERF28	0.008817	0.03489	0.252713
TkERF17/ TkERF85	0.008811	0.04943	0.178247
TkERF18/ TkERF39	0.005015	0.015095	0.332209
TkERF18/ TkERF24	0.091125	0.124866	0.729785
TkERF19/ TkERF28	0.001566	0.018266	0.085721
TkERF24/ TkERF39	0.087706	0.125062	0.701307
TkERF28/ TkERF85	0.004388	0.042314	0.10369
TkERF28/ TkERF94	0.008816	0.056664	0.155575
TkERF30/ TkERF82	0.394187	0.54574	0.722298
TkERF35/ TkERF37	0.021245	0.038706	0.548875
TkERF41/ TkERF43	0.182809	0.341772	0.534886
TkERF52/ TkERF57	0.005816	0.014883	0.390797
TkERF62/ TkERF93	0.323095	2.316687	0.139464
TkERF64/ TkERF69	0	0.004739	0
TkERF67/ TkERF88	0.003011	0.008584	0.350724
TkERF85/ TkERF94	0.00699	0.045533	0.153514
TkAP2-7/ TkAP2-36	0.044531	0.058211	0.765
TkAP2-8/ TkAP2-32	0.00224	0.005147	0.435278
TkAP2-10/TkAP2-17	0.002468	0.011785	0.209416
TkAP2-25/ TkAP2-32	0.014136	0.033965	0.41618
TkRAV3/ TkRAV4	0.003766	0.004658	0.808367

**Table 2:** Ka, Ks and Ka/Ks values for the AP2/ERF genes in Trichosanthes and Arabidopsis

Orthologous pairs	Ka	Ks	Ka/Ks
TkERF2/AT1G78080.1	0.311575	1.659059	0.187802
TkERF3/AT1G78080.1	0.346134	1.462689	0.236643
TkERF10/AT1G78080.1	0.245749	2.276362	0.107957
TkERF19/AT4G17500.1	0.316265	1.637429	0.193148
TkERF25/AT5G51990.1	0.361576	1.537269	0.235207
TkERF48/AT4G17500.1	0.316265	1.737577	0.182015
TkERF49/AT3G23240.1	0.31717	1.471628	0.215523
TkERF53/AT1G64380.1	0.46312	2.064286	0.224349
TkERF60/AT1G78080.1	0.333777	1.461567	0.228369
TkERF63/AT1G78080.1	0.235316	2.695244	0.087308
TkERF84/AT3G23240.1	0.31717	1.471628	0.215523
TkAP2-2/AT4G37750.1	0.366574	2.421057	0.151411
TkAP2-6/AT2G41710.1	0.202289	1.874449	0.107919
TkAP2-17/AT5G57390.1	0.2386	2.381813	0.100176
TkAP2-27/AT4G37750.1	0.300933	1.888938	0.159313
TkRAV1/AT1G68840.1	0.307604	2.181663	0.140995
TkRAV1/AT1G25560.1	0.348257	2.389618	0.145738

### Gene ontology (GO) annotation analysis of *TkERF* genes

**Fig. 3:**
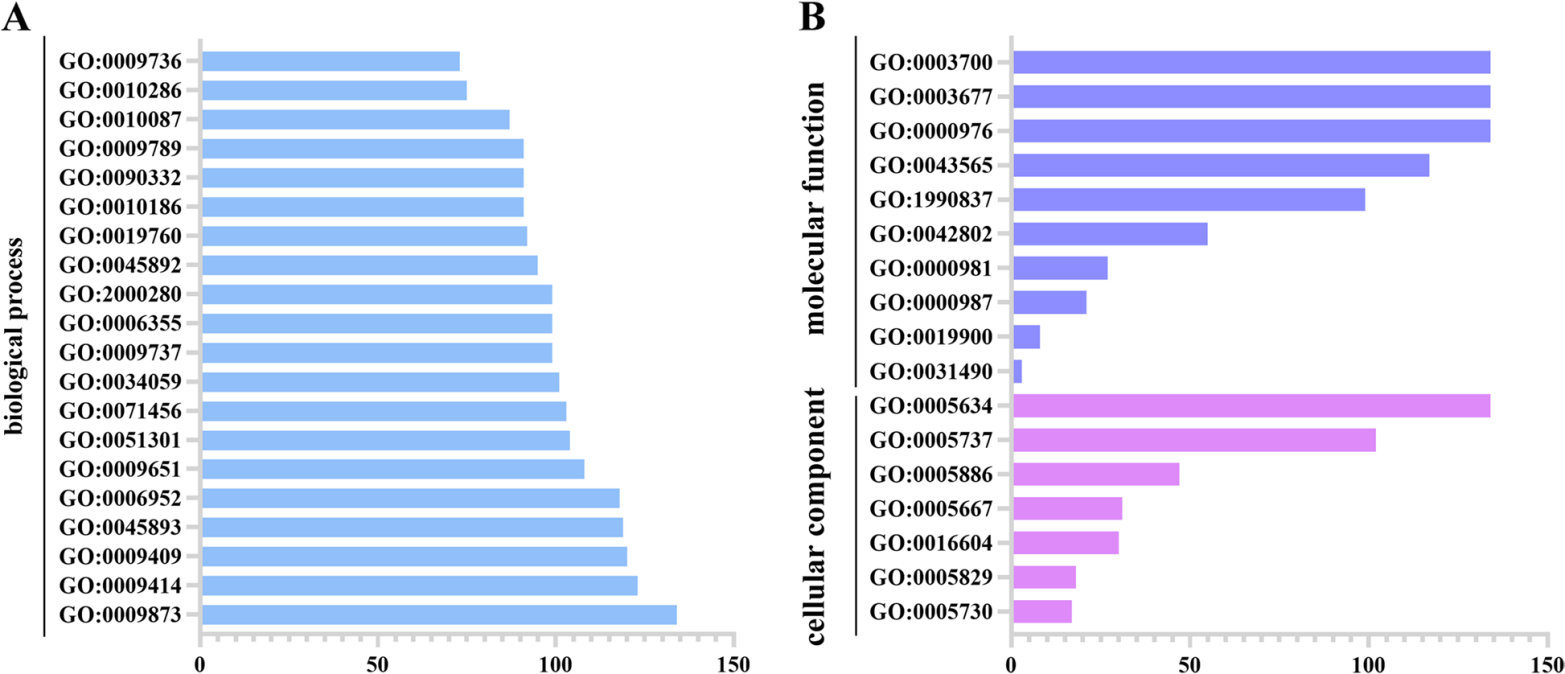
Gene Ontology (GO) results in TkAP2/ERF proteins. Different colored representdifferent function

A total of 135 genes were annotated by TBtools software, except TkERF46 had no annotation information. 181 GO terms were annotated in total, including 164 categories in biological process, 7 categories in cellular component and 10 categories in molecular function (Table S2). The top 20 terms of biological process, all terms included in the cellular component and the molecular function were analyzed (Fig. 3). Under biological process, most genes were predicted to be involved in hormone and stress response. For examples, 134 genes were predicted to involved in ethylene-activated signaling pathway (GO:0009873), 99 genes were predicted to be respond to abscisic acid (GO:0009737), 120 genes were predicted to be respond to cold (GO:0009409) and 108 genes were predicted to be respond to salt stress (GO:0009651). Under cellular component, 134 and 102 genes were assigned to nucleus (GO:0005634) and cytoplasm (GO:0005737), respectively, only 17 genes were assigned to nucleolus (GO:0005730). Among the molecular function analyses, 134 genes were found to be associated with transcription cis-regulatory region binding (GO:0000976), DNA binding (GO:0003677) and DNA-binding transcription factor activity (GO:0003700), only 3 genes were related to chromatin DNA binding (GO:0031490).

### Expression analyses of *TkERF* genes during flowering stage

**Fig. 4:**
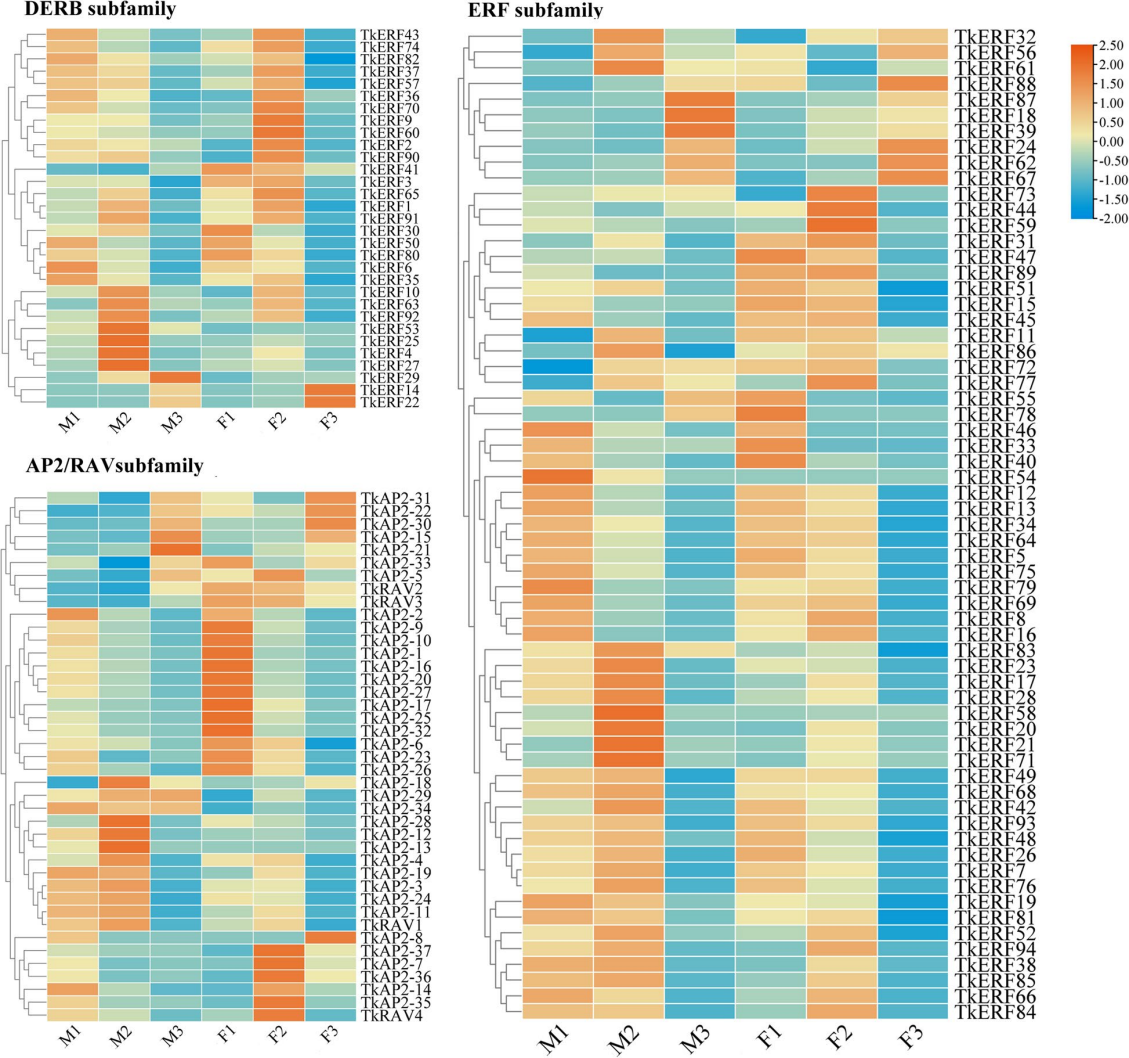
Heat map of *TkAP2/ERF* genes at different flowering stages based on transcriptome data. M1, male buds; M2, male preliminary bloom; M3, male full-bloom; F1, female buds; F2, female preliminary bloom; F3, female full-bloom

ERF transcription factors play roles in plant hormone signaling pathway and have an impact on plant growth. Studies showed the content of plant hormones in male and female flowers were different [19]. In order to analyze the ERF expression changes during flowering stage, 135 *TkERF* genes Fragments Per Kilobase of exon model per Million mapped reads (FPKM) were counted (Table S3) and showed in Fig. 4. 118 genes with FPKM value > 1 in one or more groups were selected for further study. The expression changes of the same sex flowers (female and male flowers) at different flowering stages and the different sex flowers (using the male flower for reference) at the same stage were compared (Fig. S1). For male flowers, the expression levels of 15 genes were increased, while only the expression level of *TkERF27* increased in preliminary bloom and then decreased in full bloom. Nineteen genes were both differentially expressed at both male and female flowering stage. TkERF7, TkERF66, TkERF50, TkERF68, TkAP2-1, TkAP2-9, TkAP2-10, TkAP2-20 and TkAP2-24 were down-regulated, and TkERF14, TkERF22, TkERF24, TkERF39, TkERF62, TKERF67, TkAP2-7, TkAP2-21, TkAP2-22 and TkAP2-30 were up-regulated. For female flowers, fifty-two *TkERF*s were difference expressed genes (DEGs), including 32 down-regulated and 20 up-regulated. The genes expression levels fold change (FC) of female flowers compared to male flowers was calculated, only 7 DEGs were identified, *TkERF2*, *TkERF20*, *TkERF90*, *TKAP2-34* and *TKAP2-25* were down-regulated, while *TkERF56* and *TkRAV2* were up-regulated.

### Co-expression modules of TkERFs and genes related hormone signal pathway

**Fig. 5:**
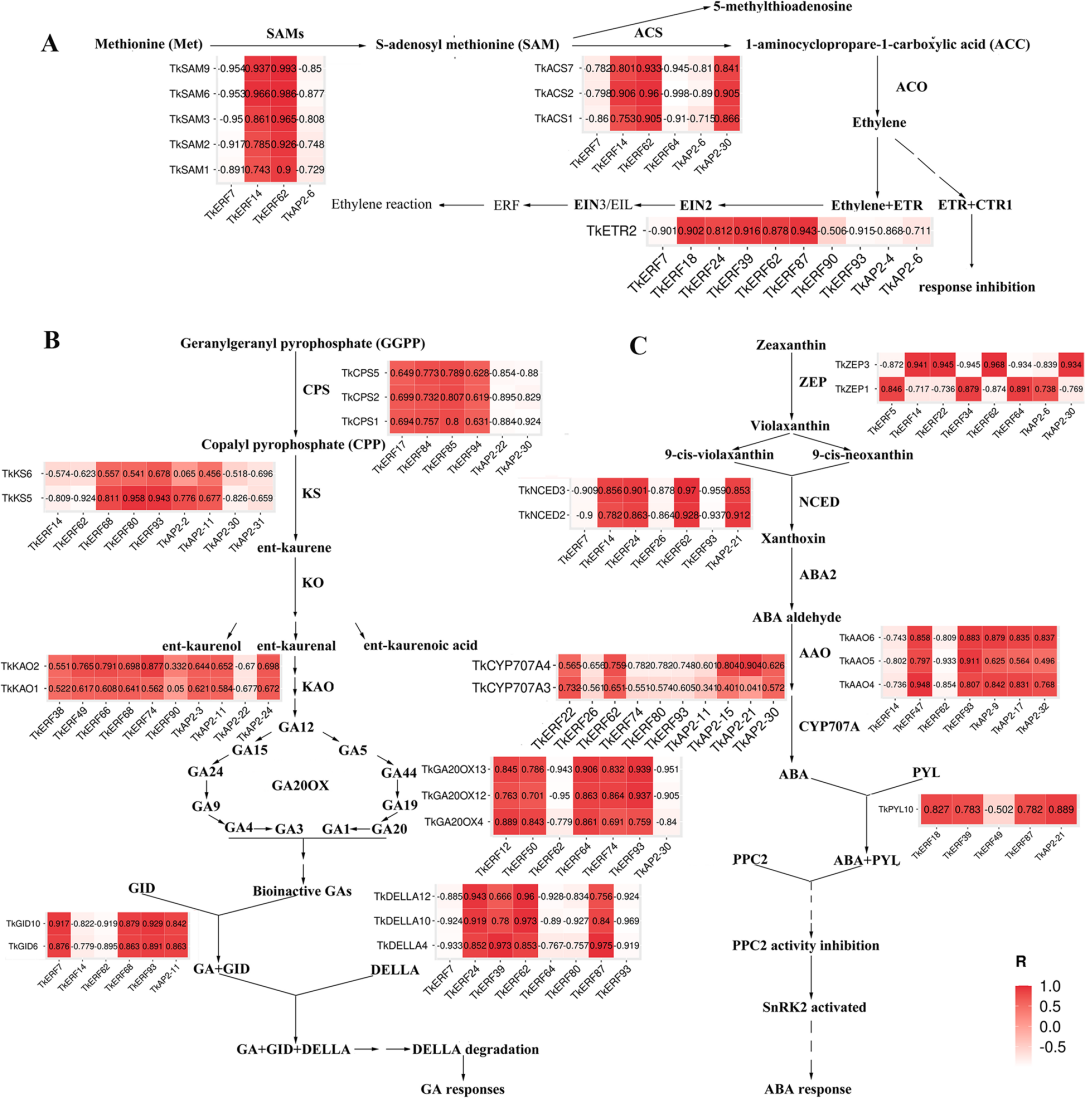
Schematic diagram of ERF involvement ETH (**A**), GA3 (**B**) and ABA (**C**) signaling pathway during flowering. The correlation coefficients between hormone pathway related genes and ERF genes were calculated by R software. The pairs whose *p*-value was ≥ 0.05 was deleted, co-expression networks were performed using the OmicStudio tools, R stands for correlation coefficient. For each gene, the gene pairs with the top 10 values were selected for for mapping

To preliminarily investigate the potential involvement of TkERFs in ETH, ABA and GA signaling pathways, we identified the DEGs of *TkERFs* and three hormone signals pathways during the male and female flower growth stages Pearson correlation coefficient (PCCs) were calculated to analyze the relevance between *TkERFs* and genes involved in the three plant hormone signaling pathways. As shown in Fig. 5 and Fig. S2, 12 DEGs (*TkSAM1*, *TkSAM2*, *TkSAM3*, *TkSAM4*, *TkSAM6*, *TkSMA8*, *TkSAM9*, *TkACS1*, *TkACS2*, *TkACS7*, *TkACS9* and *TkACS11*) involved in ethylene synthesis pathway and one DEG (*TkETR2*) involved in ethylene transduction pathway showed connections to 50 *TkERF* DEGs; for GA signaling pathways, 22 DEGs (TkCPS1, TkCPS2, TkCPS4, TkCPS5, TkKS1, TkKS5, TkKS6, TkKAO1, TkKAO2, TkGA20OX12, TkGA20OX13, TkGA20OX14, TkGA20OX15, TkGA20OX16, TkGA20OX18, TkGA20OX2, TkGA20OX3, TkGA20OX4, TkGA20OX5, TkGA20OX6, TkGA20OX7 and TkGA20OX8) related to GA synthesis pathway and 6 DEGs (TkDELLA10, TkDELLA11, TkDELLA12, TkDELLA3, TkDELLA4 and TkDELLA9) related to GA transduction pathway showed connections to 64 TkERF DEGs; for ABA synthesis and transduction pathways, 16 genes (TkNCED1, TkNCED2, TkNCED3, TkNCED4, TkNCED5, TkNCED6, TkNCED7, TkZEP1, TkZEP3, TkAAO1, TkAAO4, TkAAO5, TkAAO6, TkAAO9, TkCYP707A3 and TkCYP707A4) participated in ABA synthesis and two genes (TkPYL2 and TkPYL10) participated in ABA transduction, showed connections to 57 TkERF DEGs.

### Expression analysis of *Trichosanthes kirilowii* AP2/ERF genes by qRT-PCR

Based on the transcriptome sequence and co-expression network results, the expression levels of twenty-three *TkAP2/ERF* genes were selected, and their relative expression levels in four tissues (root, stem, leaves and flowers) were analyzed. As shown in Fig. S3, most genes were expressed relatively high in leaves. The leaves were selected as samples to extract RNA for qRT-PCR experiment. Previous studies reported that AP2/ERF transcription factor families were involved in several plant hormone signaling pathways and abiotic stress responses [2]. To investigate the potential functions of TkAP2/ERF genes, we conducted qRT-PCR to examine candidate gene expression after 4°C, NaCl, PEG, ABA, Ethylene, and GA3 treatments. Primers used for qRT-PCR were listed in Table S5.

**Fig. 6:**
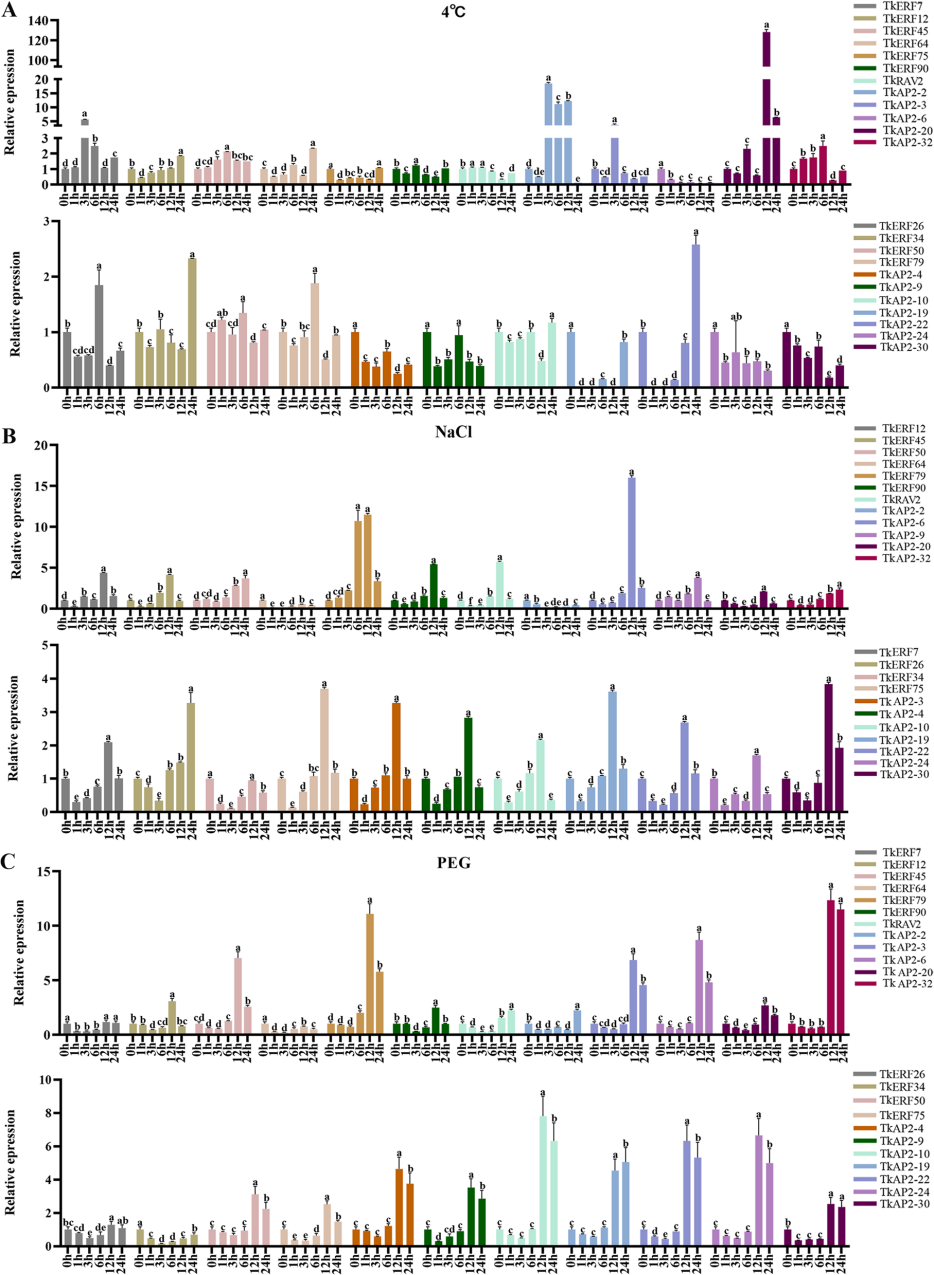
Expression patterns of 23 *TkAP2/ERF* genes in response to various stresses treatments at 0, 1, 3, 6, 12 and 24 h. **A** 4℃; **B** NaCl; **C** PEG. Y-axis: relative expression levels; X-axis: the time course of stress treatments; Error bars, 6 ± SE. Significant differences were determined by one-way ANOVA, Duncan’s multiple range test *P* < 0.05. The Mean values and standard deviations (SDs) were obtained from three biological

For 4℃ treatment, 6 genes were up-regulated with fold > 1 but < 2, 9 TkAP2/ERF genes were significantly up-regulated (fold > 2). Only *TkERF50* did not show significantly change compared to untreated leaves, and the expression levels of the remaining 7 genes were significantly down-regulated (Fig. 6A). With NaCl treatment, the expression levels of 20 TkAP2/ERF genes were up-regulated, the remaining three genes were significantly down-regulated (Fig. 6B). *TkAP2-24*, *TkERF45*, *TkAP2-3*, *TkAP2-4*, *TkAP2-10* and *TkAP2-20* had similar expression patterns. Their expression levels were the highest at 12 h, and the expression levels at 24 h were significantly down-regulated compared to 0 h. With PEG treatment, the expression levels of 19 genes were significant up-regulated, 17 genes among of them were up-regulated at 12 and 24 h, *TkERF12* and *TkERF90* were up-regulated at 12 h but down-regulated at 24 h. *TkERF7* and *TkERF26* was up-regulated with fold > 1 but < 2, while *TkERF34* and *TkERF64* were significantly down-regulated (Fig. 6C).

**Fig. 7:**
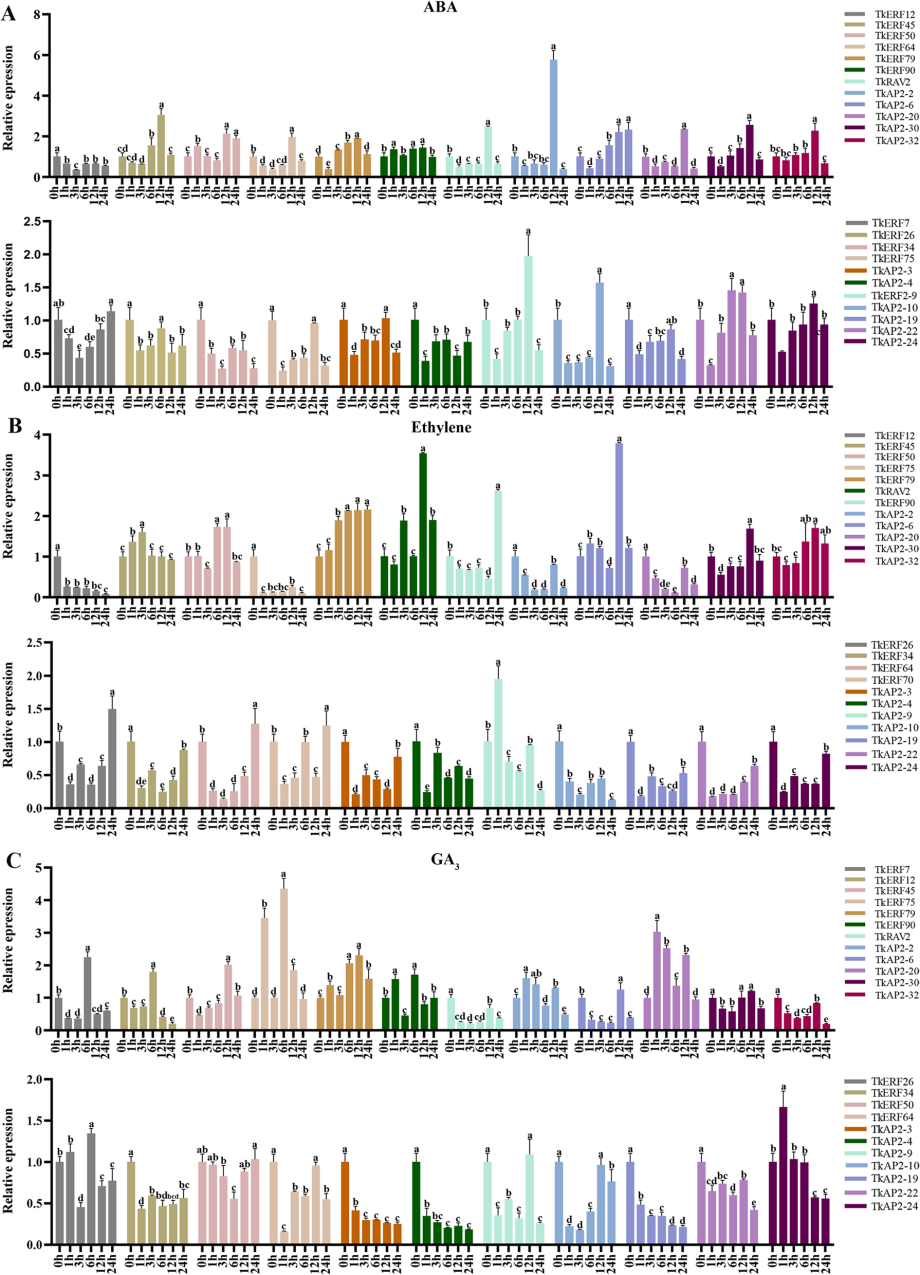
Expression patterns of 23 *TkAP2/ERF* genes in response to hormone treatments at 0, 1, 3, 6, 12 and 24 h. **A** ABA; **B** ETH; **C** GA3. Y-axis: relative expression levels; X-axis: the time course of stress treatments; Error bars, 6 ± SE. Significant differences were determined by one-way ANOVA, Duncan’s multiple range test *P* < 0.05. The Mean values and standard deviations (SDs) were obtained from three biological

For ABA treatment, the expression levels of 12 genes were up-regulated, eight of these were significantly up-regulated, with the remaining four were up-regulated with fold > 1 but < 2. Among the 8 genes with significantly up-regulated, the expression level was the highest at 12 h except for *TkAP2-6*, while its expression level at 24 h was still increased (Fig. 7A). *TkERF12*, *-26*, *-34*, *-75*, *TkAP2-4* and *TkAP2-10* were significantly down-regulated with ABA treatment. With ETH treatment, a total of 12 genes expression levels were up-regulated, and only 4 genes (TkERF79, TkERF90, TkRAV2 and TkAP2-6) were significant up-regulated (fold > 2). The expression level of TkERF90 was almost unchanged from 1–12 h and significantly increased at 24 h, while the other three genes were almost continuously increased at 0–24 h (Fig. 7B). Under ETH treatment, the expression levels of 11 genes decreased significantly. The expression levels of 14 TkAP2/ERF genes were up-regulated after GA3 treatment, while only *TkERF7*, *-45*, *-75*, *-79* and *TkAP2-20* were significantly increased (fold > 2). For these genes, the expression levels of TkERF7 and TkERF75 were highest at 6 h, while the remaining three genes had the highest expression levels at 12 h (Fig. 7C).

### Co-regulatory networks of *TkAP2/ERF* genes under abiotic stress and hormone treatments

**Fig. 8:**
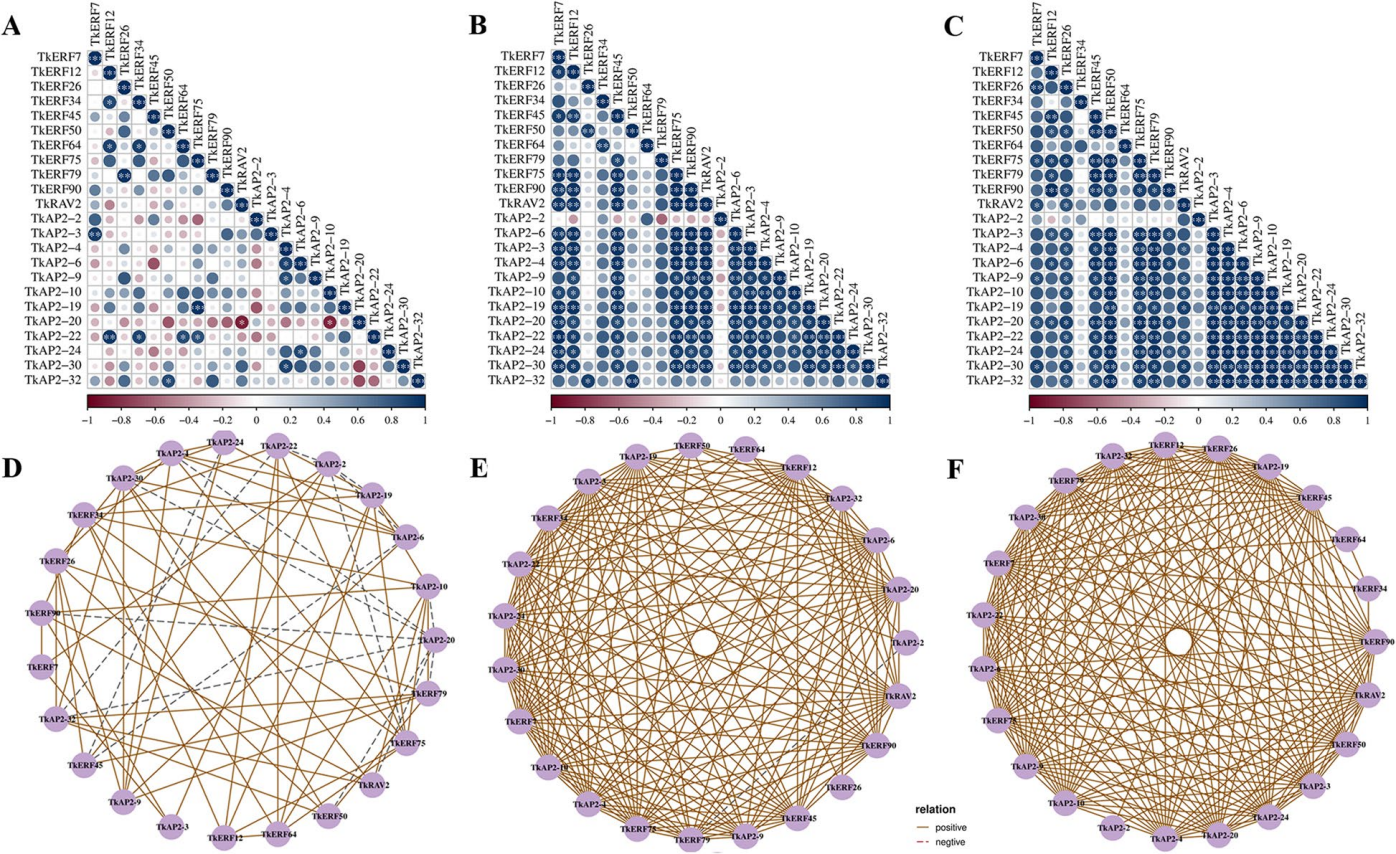
Correlations and co-regulatory networks of *TkAP2/ERF* genes under 4 ℃, NaCl and PEG treatment. Correlation analysis of *TkAP2/ERF* genes under 4 ℃ (**A**), NaCl (**B**) and PEG (**C**) treatment was performed based on the PCCs of gene pairs calculated using the R software package. Correlations are indicated by the size and colour of circles. The lower bar represents the PCC values. *, ** and *** represent correlations with *p*-value ≤ 0.05, *p*-value ≤ 0.01 and *p*-value ≤ 0.001, respectively. The coregulatory network of *TkAP2/ERF* under 4 ℃ (**D**), NaCl (**E**) and PEG (**F**) treatment was illustrated by Cytoscape. The significant PCCs of gene pairs (*p*-value ≤ 0.05) are included, and different line colors and styles indicate the different kinds of relation

**Fig. 9:**
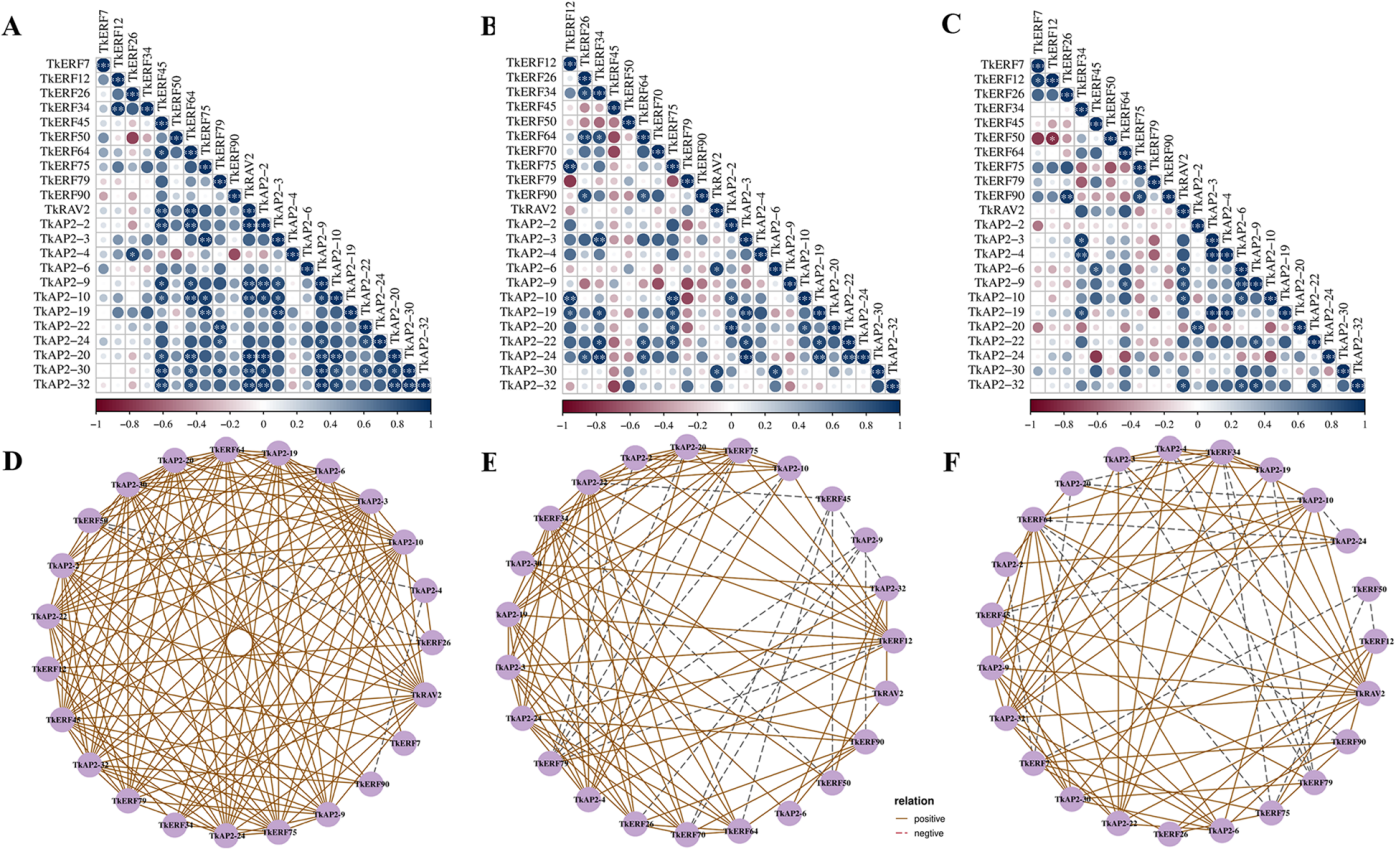
Correlations and co-regulatory networks of *TkAP2/ERF* genes under ABA, ETH and GA3 treatment. Correlation analysis of *TkAP2/ERF* genes under ABA (**A**), ETH (**B**) and GA3 (**C**) treatment was performed based on the PCCs of gene pairs calculated using the R software package. Correlations are indicated by the size and colour of circles. The lower bar represents the PCC values. *, ** and *** represent correlations with *p*-value ≤ 0.05, *p*-value ≤ 0.01 and *p*-value ≤ 0.001, respectively. The coregulatory network of *TkAP2/ERF* under ABA (**D**), ETH (**E**) and GA3 (**F**) treatment was illustrated by Cytoscape. The significant PCCs of gene pairs (*p*-value ≤ 0.05) are included, and different line colors and styles indicate the different kinds of relation

To investigate the connections among these genes in response to 4 ℃, PEG, salt, ABA, ETH and GA3, the co-regulatory networks were established based on the PCC data with relative expression levels were established (Figs. 8 and 9). With 4 ℃ treatment, many genes were positively correlated, only *TkAP2-2* and *TkERF75/AP2-6/-19/-22*, *TkAP2-20* and *TkRAV2/TKERF50/79/90/AP2-4/-10/-30/-32*, *TkERF45* and *TkAP2-6/-24*, *TkAP2-22* and *TkAP2-32* had a negative correlation. (Fig. 8A and D). Under NaCl treatment, only *TkAP2-2* and *TkERF79* showed a negative correlation (Fig. 8B and E). And through PEG treatment, all the 23 genes appeared to have different degrees of positive correlation (Fig. 8C and F). Three gene pairs showed negative correlations with ABA treatment, including *TkERF26* and *TkERF50*, *TkAP2-4* and *TkERF50*, *TkAP2-4* and *TkERF90* (Fig. 9A and D). Under ETH treatment, *TkERF79* and *TkERF12/-75/AP2-2/-10/-20*, *TkERF45* and *TkERF50/-64/-70/AP2-22/-32*, *TkAP2-9* and *TkERF26/-70/-79/-90*, *TkERF34* and *TkERF50* were negatively correlated (Fig. 9B and E). Seventeen pairs showed a negative correlation trend with GA3 treatment (Fig. 9C and F), including *TkERF34 and TkERF75/-79/AP2-20*, *TkERF50* and *TkERF12/-75*, *TkERF7* and *TkAP2-2/-10/ERF50*, *TkERF64* and *TkERF75/-90/AP2-24*, *TkERF79* and *TkAP2-3/-4*, *TkAP2-10* and *TkAP2-20/-24*, *TkAP2-19* and *TkERF79*, *TkAP2-24* and *TkERF45*.

### Subcellular localization and transcription activation assay

**Fig. 10:**
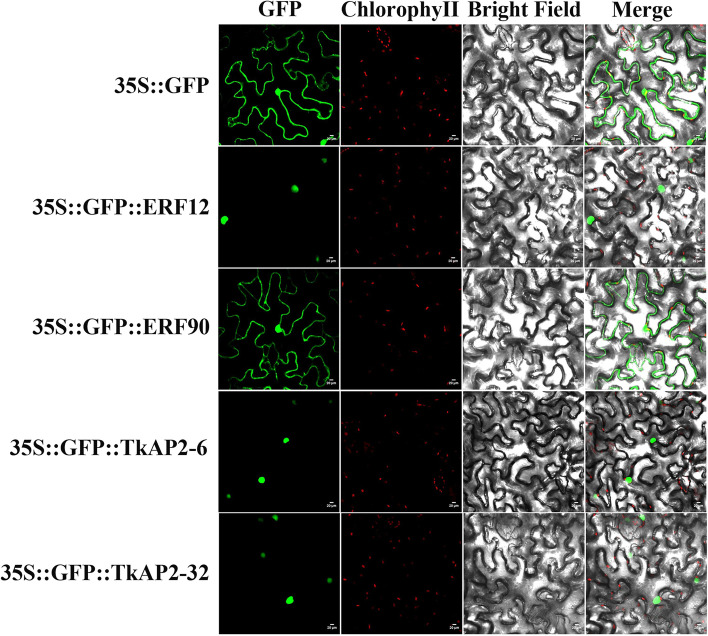
Subcellular localization of TkERF12, TkERF90, TkAP2-6 and TkAP2-32. The 35S::GFP::TkERF12 construct, 35S::GFP::TkERF90, 35S::GFP::TkAP2-6, 35S::GFP::TkAP2-32 and the control vector 1305(35S::GFP) were transformed into Nicotiana tabacum leaves, respectively. The GFP signals in cells were observed by confocal microscopy

*TkERF12*, *TkERF90*, *TkAP2-6* and *TkAP2-32* were selected for conduct subcellular localization and transcription activation assay experiments. To examine the subcellular localization of *TkERF12*, *TkERF90*, *TkAP2-6* and *TkAP2-32*, the *TkERF12-GFP*, *TkERF90-GFP*, *TkAP2-6-GFP* and *TkAP2-32-GFP* were constructed and transformed in tobacco. The observations showed that TkERF12, TkAP2-6, and TkAP2-32 were located in the nucleus, and TkERF90 was located in the nucleus and cytoplasm (Fig. 10).

**Fig. 11:**
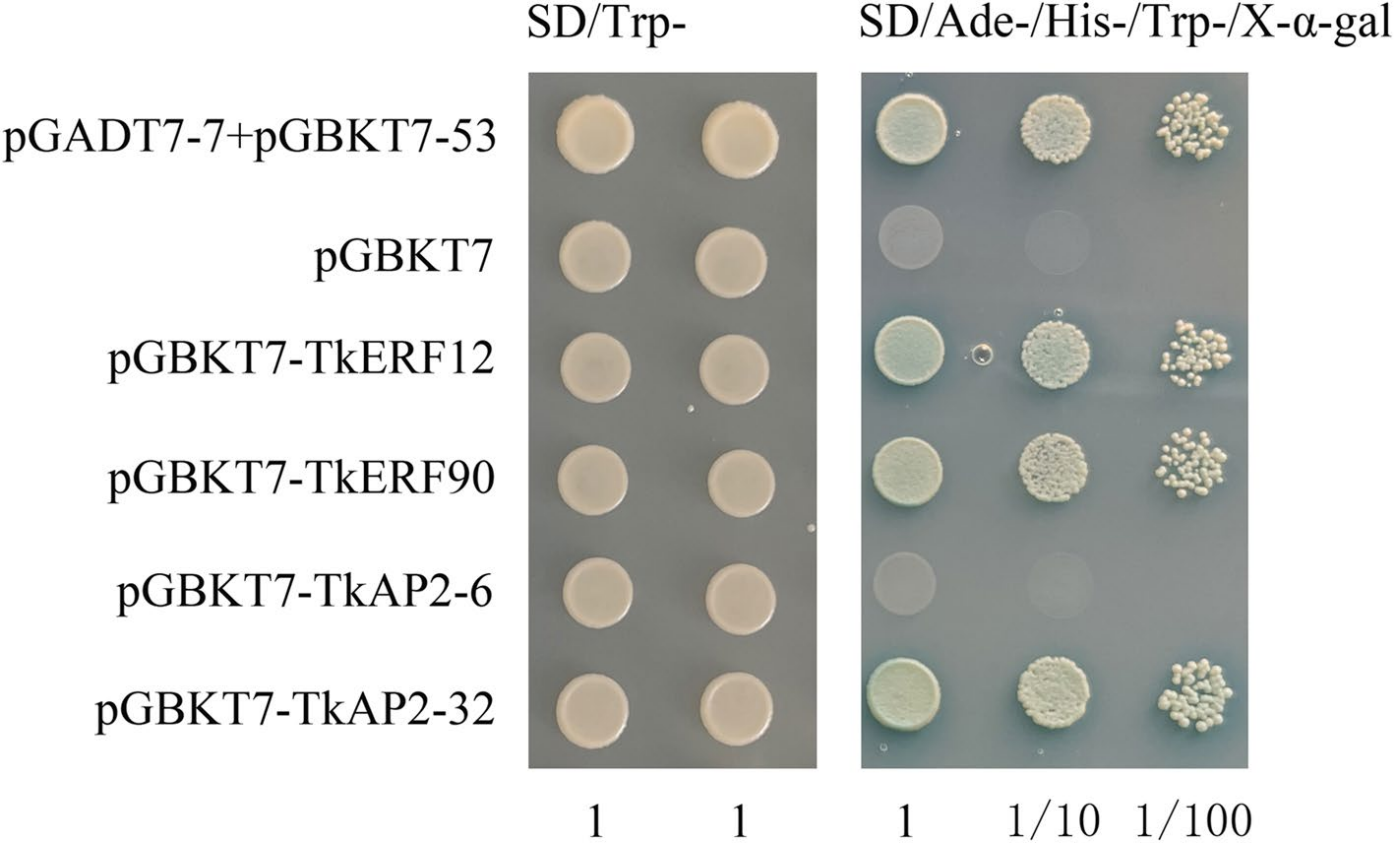
Transcriptional activity analysis of TkERF12, TkERF90, TkAP2-6 and TkAP2-32. Positive control, pGBKT7–53 and pGBKT7-T; Negative control, pGBKT7

For transcription activation assay, the yeast fusion expression vector *TkERF12-pGBKT7*, *TkERF90-pGBKT7*, *TkAP2-6*-*pGBKT7* and *TkAP2-32*-*pGBKT7* were constructed and transformed into yeast receptor cell AH109. As shown in Fig. 11, the positive control, negative control, *TkERF12-pGBKT7*, *TkERF90-pGBKT7*, *TkAP2-6*-*pGBKT7* and *TkAP2-32*-*pGBKT7* grew well on the SD/Trp-plate. And the positive control, *TkERF12-pGBKT7*, *TkERF90-pGBKT7* and *TkAP2-32*-*pGBKT7* were also able to grow well on the *SD/Trp − /His − /Ade − /X-α-gal* plate and produce a blue color reaction, while the negative control and *TkAP2-6*-*pGBKT7* can not grow on the SD/Trp − /His − /Ade − /X-α-gal plate and produce a blue color reaction. All of these proved that TkERF12, TkERF90 and TkAP2-32 had self-activating activity, while TkAP2-6 had no self-activating activity.

## Discussion

This study focuses on the *T. kirilowii* ERF gene family, which has not been previously researched. *AP2/ERF* genes were plant-specific transcription factors, and previous studies on ERF proteins in Arabidopsis [1], rice [1] and cucumber were published [31], the ERF function on abiotic stresses and plant hormone signal pathway were reported. In this study, transcriptome data was used to analyze the evolutionary relationship of TkAP2/ERF gene familiy, and qRT-PCR was conducted to study the response of genes to stress and hormone treatment. Additionally, we selected *TkERF12*, *TkERF90*, *TkAP2-6* and *TkAP2-32* for transcriptional activity and subcellular localization assays.

 The study identified 135 T*. kirilowii* AP2/ERF genes from transcriptome data, including 94 *ERF* subfamily members, 37 *AP2* subfamily members, 4 *RAV* subfamily members. Similar distribution of different subfamilies was observed in other plants. For example, cucumbers contained 131 *AP2/ERF* genes, including 109 *ERF* genes, 18 *AP2* genes, 4 *RAV* genes. Previous studies found that the number of AP2/ERF superfamily members were relatively stable and did not correlate absolutely with genome size. For instance, the numbers difference of AP2/ERF genes in Arabidopsis, maize, poplar and willow was not significant, containing 145 [1], 184 [18], 200 [32] and 173 [33] genes respectively. However, the genomes of the species had significantly different sizes, with sizes ranging from 125 Mb [34], 2.25 Gb [35], 480 Mb [36] and 1.07 Gb [37], respectively. All of these proved that the variation in the number of ERF members among different species may be caused by gene evolution and replication.Ka/Ks value confirmed that this family experienced strong purifying selection pressure during evolution [38].

The AP2/ERF superfamilies of* T. kirilowii* were categorised into 4 subfamilies including ERF, DREB, AP2 and RAV, which contained 63, 31, 37, and 4 members, respectively. This result supports the conclusion that ERF and DREB differ significantly in gene number within the same species, while RAV is a small subfamily containing few members [39–42]. The domains and motifs of transcription factors are usually associated with DNA binding, transcriptional activity and protein interactions [43]. In this study, all four members of the RAV subfamily had only 2 motifs, while theother subfamily members had a maximum of eight motif elements. The motif 1, motif 2 and motif 7 were conserved in the most *TkAP2/ERF* genes (Fig. 2). The motif 1 contained the RAYD element, which could be involved in protein–protein or protein-DNA interactions due to the amphiphilic nature of its central region α- Spiral structure [2]. The main difference between the ERF and DREB subfamilies was that two bases in motif 2 are inconsistent [4]. Motif2 of the ERF subfamily can specifically bind to the GCC-box element of the target gene promoter and participate in the response to ethylene, pathogen attack and abiotic stress, while DREB motif2 subfamily can specifically bind DRE/CRT elements and A/GCCGAC motif which were related to drought and cold response respectively and participate in plant stress response [2]. In addition, ERF B6 group included motif 5, motif 6, motif 8, motif 9 and motif 10 in addition to motif 1, motif 2 and motif 7, some genes in DREB A6 group included motif 8. Motif 3 and motif 4 are only present in the AP2 subfamily. Furthermore, the AP2 subfamily contained two motif 1, they can combine with GCAC(A/G)N(A/T)TCCC(A/G)ANG(C/T) motifs to participate in plant growth. All of these proved that differences kinds of motif in different subfamilies suggest that they may play different roles in plants. In addition, some new motifs have also evolved, which may play new functions in plants, and the functions of these new motifs need to be further verified.

Previous studies have shown that the AP2/ERF transcription factor was a key regulatory factor in different plant development processes and stress responses [44], furthermore, the two functions were also related to the hormone signaling pathway [2]. Therefore the *AP2/ERF* gene family may be considered as a potential candidate for plant improvement. In order to predict gene functions, gene expression profiles analysis can be used as a preliminary tool [45]. For plant development processes, the expression levels of ERF in male and female flowers at three flowering stages were analyzed. A number of DEGs that may be involved in plant growth were identified. For examples, *TkERF66*, a member of the ERF subfamily B1 branch, its expression levels of preliminary and full-bloom were lower than that of buding (Fig. 4). And *AtERF11* (AT1G28370) which belonged to the same branch, was proved to promote internode elongation by activating GA biosynthesis and signaling [46]. Interestinglly, the *TkERF66* also showed a connection with genes in GA signaling pathway (Fig. 5). Therefor we speculated that *TkERF66* has similar functions with *AtERF11*, that is *TkERF66* might be affect plant growth and development by involved in GA signaling pathway. The *TkERF14* and *TkERF22* belong to the DREB-A5 clan (Fig. 2), and the *AtDEAR4* (AT4G36900) which belongs to same clan was proved to be associated with leaf growth and development and can be induced by ABA and JA [47]. Similarly, the expression levels of *TkERF14* and *TkERF22*in preliminary and full-bloom were higher than that of buding (Fig. 4), indicating their potential involvement in flowering. Additionally, these two genes also showed connection with ABA signaling pathway genes (Fig. 5). Both of this proved *TkERF14* and *TkERF22* have similar function with *AtDEAR4*. The mutation of *T. kirilowii* male plants into monoecious plants was common in nature, but the mechanism of sex differentiation remains unclear.. In some model plants such as cucumber, ERF genes were showed to influence plant sex differentiation by participating in plant hormone signaling pathways [18, 19]. In this study, the expression levels of ERF in the female and male flowers buds were compared, and seven differentially expressed genes were identified, namely *TkERF2*, *TkERF20*, *TkERF56*, *TkERF90*, *TkAP2-34*, *TkAP2-35* and *TkRAV2* (Fig. S1). *TkERF20* and *AtERF8* (AT1G53170) belong to the same subfamily, while *AtERF8* was showed to be involved in ABA signaling [48]. *TkERF56* belongs to the ERF-B2 branch, and *AtERF12* (At3G16770), a gene in this branch, was showed to be involved in the ethylene signaling pathway in Arabidopsis [49]. We suspected that TkERF20 and TkERF56 genes are involved in plant signaling pathways, whether these genes were involved in the hormonal signaling pathway in *T. kirilowii* and whether they affect sex differentiation need further study.

Tissue expression patterns showed that the most *TkERF* genes were expressed highest in the leaves (Fig. S3), therefore, the leaves were used for the qRT-PCR gene expression analysis. In this study, based on the transcriptome sequence and co-expression analysis, twenty-three *TkAP2/ERF* genes were selected to investigate whether they were responded to abiotic stress and hormonal treatment. 4 ℃ treatment significantly induced the expression of 15 TkAP2/ERF genes, among these genes, *TkAP2-2*, *TkAP2-3* and *TkAP2-30* showed a particularly significant up-regulated (Fig. 6), indicating that these three genes may be more sensitive to low temperature stress. Furthermore, ETH, GA and ABA were considered to be an important regulatory hormone in plant low temperature response [2], while *TkAP2-2*, *TkAP2-3* and *TkAP2-30* were only responded to ABA treatment and are all highly expressed at 12 h. Therefore, we speculate that these three genes may respond to low temperature stress through ABA signaling pathway. ERF genes were showed responding to salt stress in many species, such as *AtERF1* [2], *OsERF106* [50] and *TgERF1* [51]. In *T. kirilowii*, we found that *TkERF5*0 and *TkERF90* genes, belonging to the DREB subfamily, were responded to salt stress, and were both significantly up-regulated at 12 h (Fig. 6). This indicates that salt has a significant inducement effect on DREB subfamily members, which was consistent with previous studies [2]. Furthermore, the *TkERF50* responded to ABA treatment, while the *TkERF90* responded to ETH treatment. It is speculated that salt tolerance of *TkERF50* and *TkERF90* may be related to hormonal signaling pathways. Some members of AP2 subfamily were confirmed to be involved in salt tolerance [51]. The study found that *TkAP2-6* and *TkAP2-9* expression levels were significantly higher at 12 h under NaCl treatment (Fig. 6), and were also responsive to ABA and ETH treatment (Fig. 7,). For PEG treatment, we observed increased expression levels of members in the ERF, DREB and AP2 subfamilies.. For examples, DREB-A2 and A6 groups were both proved to be involved in plant drought [42], and *TkERF50* and *TkERF90* which belong to the two groups, respectively, responsive to PEG treatment (Fig. 6). The Arabidopsis RAV1 and RAV2 transferred into cotton can increase fiber length and obtaining the same yield under drought stress compared to control conditions [2]. In this study, the expression level of *TkRAV2* increased significantly after 24 h of drought treatment (Fig. 6), and similar to *TkERF90*, it also responded to ABA and ETH treatments (Fig. 7). So we speculated that TkRAV2 may play a role in drought stress in plants.

## Conclusions

In this study, 135 full-length AP2/ERF genes were identified and classified into four subfamilies. Phylogenetic comparison and homology analysis were used to determine the evolutionary characteristics of *TkAP2/ERF*. The gene expression of *TkAP2/ERF* were also analyzed to identify potential target genes, revealing that TkAP2/ERF gene played roles in flowering and responded to abiotic stress. In conclusion, these results provide a reference for understanding the individual biological role of *TkAP2/ERF* genes in *T. Kirilowii*.

## Materials and methods

### Identification of the *ERF* gene family in *T. kirilowii*

The transcriptome data used in this study were uploaded to the National Center for Biotechnology Information (NCBI) website (project accession number PRJNA858494). Genes containing AP2 domain (PF00847) were screened from Pfam annotation files [38], and the CDS and protein sequences were extracted from transcriptome sequencing data. HMM profile in the Pfam database (http://pfam.janelia.org/search/sequence) [52], SMART (http://smart.embl-heidelberg.de/) databases and NCBI conservative domain search (http://www.ncbi.nlm.nih.gov/Structure/cdd/wrpsb.cgi) were used to search for whether these candidate genes contained a complete domain. The genes without a complete AP2 domain were removed, genes containing one AP2 domain were identified as the ERF or DREB subfamily, further differentiation was made according to the conservative domain; genes containing two AP2 domains were identified as the AP2 subfamily, and genes containing one AP2 domain and a complete B3 domain (PF02362) were identified as the RAV subfamily [3]. The genes’ amino acids number, ORF length, pI and Mw were calculated on ExPASy website (http://www.expasy.ch/tools/pi_tool.html) [53]. WOLF PSORT (http://www.genscript.com/psort.html) website was used to predict the subcellular localization of the TkAP2/ERF proteins [54].

### Phylogenetic tree construction and conserved motifs analysis of TkERF gene family members

Arabidopsis genome sequences were obtained from the Arabidopsis Information Resource (http://www.arabidopsis.org) and AtERF numbers were downloaded from the NCBI website (https://www.ncbi.nlm.nih.gov/pmc/articles/PMC1361313/) [1]. MEGA 11.0 software was used to construct an un-rooted phylogenetic tree by Neighbor-Joining method, and the replications was set as 1000 [55].

TkERF conserved motifs were identified on the MEME online tool (http://meme.sdsc.edu/meme/intro.html) [56]. The parameters were set as follows: num of motifs to find = 10, min motif width = 6, max motif width = 200.

### Homologous pairs identify and Ka/Ks values calculate

Paralogous pairs in *T. kirilowii*, orthologous pairs between *T. kirilowii* and *A. thaliana* were identified according to the method described in Blanc and Wolfe, 2004. The genes with nucleotide length shorter than 300bp were deleted from the homologous pairs, the sequence similarity of two genes in a homologous pair must be greater than 40%. The alignment of the pair was performed by MEGA 11.0 software. Then the alignment results were submitted to DnaSP 5 software to count Ks and Ka rates [57]. As a rule, the value of Ka/Ks indicated the kinds of selection, Ka/Ks < 1 indicated negative selection, Ka/Ks = 1 indicated neutral evolution, Ka/Ks > 1 indicated positive selection [58, 59].

### GO annotation analysis

The TkERF protein sequences were blast in NNCBIwebsite (https://www.ncbi.nlm.nih.gov/), and the UniProKB/Swiss-Prot (Swissprot) was selected as the database. The GO annotation analysis was conducted using TBtools software [60], the parameters were set as following: MaxEvalue, 1e-5, MinWeightCov, 0.33.

### Expression pattern and co-expression network analysis of TkERF genes in different flowers periods

The FC and significant q-values were calculated by TBtools software [60]. The gene which met the following conditions were defined as DEGs: FC > 1 and *p*-value < 0.05. The FPKM change values of TkERFs were used for heat map drawing.

The gene related to ETH, ABA and GA3 signal pathway were first screened using the PF number, and then the sequences of the candidate genes were submitted to NCBI Conserved Domains website (https://www.ncbi.nlm.nih.gov/cdd/?term=) to verify whether they contained complete conserved domain. The correlation coefficients between these genes and ERF genes were calculated by R software. The pairs whose p-value was ≥ 0.05 was deleted, co-expression networks were performed using the OmicStudio tools (https://www.omicstudio.cn/index).

### Plant materials, growth conditions, stress treatments

*T. kirilowii* tissue culture seedlings were preserved in Institute of Horticulture, Anhui Province, China. The seedlings were cultured at 28℃ in an artificial climate chamber with 16/8 h of light/dark. The 6-week-old *T. kirilowii* seedings were used for hormone treatments and abiotic stress to compare the relative expression levels of candidate genes. For hormone treatment, the seedings were treated with 100 µM ABA solution, 100 mg L − 1 ETH and 60 ppm GA3 [61, 62]. For abiotic stress, tissue culture seedlings were treated with 100 mM NaCl, 20% PEG-6000 solution and placed at 4° C to simulate salt, drought and cold stress, respectively. All leaves were harvested at 0, 1, 3, 6, 12 and 24 h after treatment. For tissue expression pattern, six-month-old seedlings including roots, stems, leaves and flowers were collected. The samples were frozen in liquid nitrogen and stored at -80 ℃ for RNA extraction.

### RNA extraction and qRT-PCR analysis

The Spin Column Plant Total RNA Purification Kit (Shanghai, Sangon) was used to extract total RNA from roots, stems, leaves and flowers according to the manufacturer’s instructions. 1% agarose gel electrophoresis was used to detected the integrity of RNA. The concentration of purified RNAs were detected by NanoDrop 2000 spectrophotometer (ThermoFisher Scientific, Wilmington, Delaware, USA). The first strand of cDNA was synthesized using UnionScript First-stand cDNA Synthesis Mix according to the manufacturer’s instructions (Beijing, Genesand Biotech Co.,Ltd). qRT-PCR was performed on a 10 μl reaction system including 5 μl 2 × GS AntiQ qPCR SYBR Master Mix, 0.4 μl of each specific primer, 1 μl diluted cDNA template and 3.2 μl ddH_2_O. The reaction was two-step amplification: 95 ℃ predenaturation for 1 min, 40 cycles of 95 ℃ transsexual for 20 s and 60℃ annealing/extension for 30 s. The 2^−ΔΔCT^ method was used to calculate the relative expression level of each gene [60], and taking the expression in 0 h and root as a reference, set them to 1. GAPDH was used as an internal control [63], GraphPad software was used for statistical analysis [64].

### Pearson correlation analyses of qRT-PCR data

Based on the results of qRT-PCR, PCCs) and *p*-values of TkAP2/ERF gene expression levels were calculated by R software package. All gene pairs with a PCC greater than 0.5 and p-value lower than 0.05 were collected for gene co-regulatory network analysis. Using PCCs of these gene pairs, graphic visualization of the co-expression network was performed using Cytoscape [65].

### Statistical analysis

Significant differences were determined by one-way ANOVA, Duncan’s multiple range test *P* < 0.05. The Mean values and standard deviations (SDs) were obtained from three biological.

### Subcellular localization and Transcription activation assay

The full-length CDS of four candidate genes were cloned from *T. kirilowii*, and the products were cloned into the *pCambia1305* vector (Clontech, Beijing, China) containing the CaMV35S promoter and the GFP gene. The successfully constructed plasmid was transformed into Agrobacterium competent GV3101 (Shanghai Weidi Biotechnology Co., Ltd) by freeze–thaw method. The suspension was transiently transformed into tobacco leaves by injection method. Meanwhile, the pCambia1305 containing only constitutive GFP were used as control vector. The green fluorescence signal was observed by laser confocal microscope [66].

The four candidate genes were cloned into the *pGBKT7-7*, the *pGBKT7–53* and *pGBKT7-T* were used as positive control, the *pGBKT7-7* was used as negative control. The controls and constructed plasmid were transformed into Y2H-Gold (yeast competent cells) (Shanghai Weidi Biotechnology Co., Ltd). The transformed competent cells were coated on single-deficient (SD/Trp −) and triple-deficient (SD/Trp − /His − /Ade − /X-α-gal) deficient medium to observe the phenotype.

## Supplementary Information

**Additional file 1:** **Table S1.** List of 135 AP2/ERF genes identified in *Trichosanthes kirilowii* and their sequence characteristics.

**Additional file 2:** **Table S2.** Gene ontology (GO) annotation analysis of 135 *Trichosanthes kirilowii* *AP2/ERF *proteins.

**Additional file 3:** **Table S3.** Fragments Per Kilobase of exon model per Million mapped fragments (FPKM) values of 135 *Trichosanthes kirilowii* *AP2/ERF *genes.

**Additional file 4:** **Table S4.** FPKM values of genes associated with ABA, ethylene and gibberellin signaling pathways.

**Additional file 5:** **Table S5.** Primers used for the qRT-PCR analysis of TkAP2/ERF gene expression.

**Additional file 6:** **Figure S1.** The expression trends of TkAP2/ERF gene family were analyzed based on the transcriptome data of female and male flowers at different flowering stages. M1, male buds; M2, male preliminary bloom; M3, male full-bloom; F1, female buds; F2, female preliminary bloom; MF3, female full-bloom.

**Additional file 7:** **Figure S2.** Co-expression networks based on transcriptome data of female and male flowers at different flowering stages. A: Co-expression network diagram of TkAP2/ERF differentially expressed gene and ethylene signaling pathway differentially expressed gene; B: Co-expression network diagram of TkAP2/ERF differentially expressed gene and gibberellin signaling pathway differentially expressed gene; C: Co-expression network diagram of TkAP2/ERF differentially expressed gene and abscisic acid signaling pathway differentially expressed gene.

**Additional file 8:** **Figure S3.** Expression profiles of Trichosanthes *AP2/ERF* genes across different tissues by qRT-PCR.

## Data Availability

All the sequencing data were submitted to NCBI Sequence Read Archive database under accession number PRJNA858494. Arabidopsis genome sequences were obtained from the Arabidopsis Information Resource (http://www.arabidopsis.org) and AtERF number were downloaded from the NCBI website (https://www.ncbi.nlm.nih.gov/pmc/articles/PMC1361313/).
