# Supplementary material for: Transcript-wide identification and expression pattern analysis to comprehend the roles of AP2/ERF genes under development and abiotic stress in Trichosanthes kirilowii
Source: BMC Plant Biol. 2023 Jul 10;23:354. doi: 10.1186/s12870-023-04362-0 (PMC10332102; doi:10.1186/s12870-023-04362-0)
Supplement: Supplementary file 1 — Additional file 1: Table S1. List of 135 AP2/ERF genes identified in Trichosanthes kirilowii and their sequence characteristics. [file 12870_2023_4362_MOESM1_ESM.docx]

| Name | Sequence ID | ORF length (bp) | Protein | | | Subcellular localization |
| --- | --- | --- | --- | --- | --- | --- |
|  |  |  | Length (aa) | PI | MW (Da) |  |
| TkERF1 | CKF_transcript_13205 | 1096 | 365 | 5.79 | 39719.0 | nucleus |
| TkERF2 | CKF_transcript_13869 | 1102 | 367 | 5.94 | 39917.33 | nucleus |
| TkERF3 | CKF_transcript_14099 | 1096 | 365 | 5.79 | 39642.90 | nucleus |
| TkERF4 | CKF_transcript_14529 | 1078 | 359 | 6.51 | 39476.88 | nucleus |
| TkERF5 | CKF_transcript_14756 | 955 | 318 | 4.66 | 35471.33 | nucleus |
| TkERF6 | CKF_transcript_14856 | 1276 | 425 | 5.44 | 47036.37 | nucleus |
| TkERF7 | CKF_transcript_14898 | 1048 | 349 | 4.85 | 40230.21 | nucleus |
| TkERF8 | CKF_transcript_14944 | 805 | 268 | 4.68 | 30215.09 | nucleus |
| TkERF9 | CKF_transcript_15296 | 1042 | 347 | 5.50 | 38103.48 | nucleus |
| TkERF10 | CKF_transcript_15662 | 664 | 221 | 7.02 | 24324.99 | nucleus |
| TkERF11 | CKF_transcript_17114 | 1168 | 389 | 5.02 | 43243.92 | nucleus |
| TkERF12 | CKF_transcript_18347 | 1000 | 333 | 4.80 | 37387.41 | nucleus |
| TkERF13 | CKF_transcript_18377 | 922 | 307 | 8.53 | 34413.97 | nucleus |
| TkERF14 | CKF_transcript_18861 | 454 | 151 | 7.88 | 17147.04 | nucleus |
| TkERF15 | CKF_transcript_21422 | 655 | 218 | 6.73 | 24091.10 | nucleus |
| TkERF16 | CKF_transcript_21713 | 853 | 284 | 5.96 | 32479.28 | nucleus |
| TkERF17 | CKF_transcript_21797 | 610 | 203 | 8.35 | 22775.57 | nucleus |
| TkERF18 | CKF_transcript_22039 | 541 | 180 | 7.96 | 19357.43 | nucleus |
| TkERF19 | CKF_transcript_22170 | 871 | 290 | 5.30 | 31119.12 | nucleus |
| TkERF20 | CKF_transcript_22236 | 727 | 242 | 9.04 | 26616.65 | nucleus |
| TkERF21 | CKF_transcript_23084 | 883 | 294 | 5.11 | 32322.64 | nucleus |
| TkERF22 | CKF_transcript_23772 | 451 | 150 | 9.99 | 17222.42 | nucleus |
| TkERF23 | CKF_transcript_24624 | 583 | 194 | 8.78 | 21070.21 | nucleus |
| TkERF24 | CKF_transcript_26490 | 466 | 155 | 10.74 | 17221.84 | nucleus |
| TkERF25 | CKF_transcript_27023 | 637 | 212 | 5.23 | 23794.69 | nucleus |
| TkERF26 | CKF_transcript_27193 | 1147 | 382 | 5.35 | 42562.82 | nucleus |
| TkERF27 | CKF_transcript_28082 | 1075 | 358 | 6.37 | 39445.86 | nucleus |
| TkERF28 | CKF_transcript_34115 | 610 | 203 | 8.28 | 22691.51 | nucleus |
| TkERF29 | CKF_transcript_34152 | 502 | 167 | 12.00 | 18907.83 | nucleus |
| TkERF30 | CKF_transcript_34182 | 451 | 150 | 7.77 | 16487.43 | nucleus |
| TkERF31 | CKF_transcript_39083 | 1168 | 389 | 5.02 | 43248.95 | nucleus |
| TkERF32 | CKF_transcript_46628 | 1168 | 389 | 4.97 | 43284.94 | nucleus |
| TkERF33 | CKF_transcript_47277 | 643 | 214 | 8.38 | 23575.58 | nucleus |
| TkERF34 | CKF_transcript_49129 | 952 | 317 | 4.63 | 35356.15 | nucleus |
| TkERF35 | CKF_transcript_51170 | 1264 | 421 | 5.07 | 46866.12 | nucleus |
| TkERF36 | CKF_transcript_53749 | 949 | 316 | 5.09 | 35802.91 | nucleus |
| TkERF37 | CKF_transcript_56817 | 1297 | 432 | 5.12 | 48101.41 | nucleus |
| TkERF38 | CKF_transcript_56969 | 499 | 166 | 6.60 | 18727.25 | nucleus |
| TkERF39 | CKF_transcript_60735 | 538 | 179 | 8.83 | 19357.43 | nucleus |
| TkERF40 | CKF_transcript_61084 | 955 | 318 | 4.66 | 35471.33 | nucleus |
| TkERF41 | CKF_transcript_62369 | 673 | 224 | 11.23 | 25232.51 | nucleus |
| TkERF42 | CKF_transcript_63938 | 1123 | 374 | 5.27 | 41845.04 | nucleus |
| TkERF43 | CKF_transcript_66407 | 496 | 165 | 11.93 | 19222.31 | nucleus |
| TkERF44 | CKF_transcript_70234 | 1252 | 417 | 5.34 | 46277.64 | nucleus |
| TkERF45 | CKF_transcript_70802 | 919 | 306 | 4.88 | 34612.88 | nucleus |
| TkERF46 | CKF_transcript_77492 | 520 | 173 | 8.83 | 19315.50 | nucleus |
| TkERF47 | CKF_transcript_79292 | 628 | 209 | 10.28 | 24077.34 | nucleus |
| TkERF48 | CKF_transcript_79897 | 868 | 289 | 5.30 | 30958.99 | nucleus |
| TkERF49 | CKF_transcript_80455 | 682 | 227 | 4.97 | 24907.61 | nucleus |
| TkERF50 | CKF_transcript_82751 | 535 | 178 | 9.34 | 19642.21 | nucleus |
| TkERF51 | CKF_transcript_85441 | 865 | 288 | 8.24 | 32278.55 | nucleus |
| TkERF52 | CKF_transcript_85443 | 901 | 300 | 5.19 | 34151.94 | nucleus |
| TkERF53 | CKF_transcript_89259 | 922 | 307 | 8.57 | 33651.87 | chloroplast |
| TkERF54 | CKF_transcript_90092 | 919 | 306 | 4.83 | 34453.77 | nucleus |
| TkERF55 | CKF_transcript_92536 | 1168 | 389 | 5.01 | 43285.97 | nucleus |
| TkERF56 | CKF_transcript_92974 | 1168 | 389 | 5.07 | 43292.00 | nucleus |
| TkERF57 | CKF_transcript_93535 | 904 | 301 | 5.19 | 34255.13 | nucleus |
| TkERF58 | CKF_transcript_93936 | 610 | 203 | 5.46 | 22303.18 | nucleus |
| TkERF59 | CKF_transcript_97500 | 520 | 173 | 9.05 | 18233.26 | nucleus |
| TkERF60 | CKF_transcript_98907 | 1030 | 343 | 6.26 | 37276.28 | nucleus |
| TkERF61 | CKF_transcript_99056 | 781 | 260 | 5.30 | 28442.85 | nucleus |
| TkERF62 | CKF_transcript_104610 | 628 | 209 | 6.45 | 23663.91 | chloroplast |
| TkERF63 | CKM_transcript_13708 | 658 | 219 | 7.02 | 24066.60 | nucleus |
| TkERF64 | CKM_transcript_14738 | 955 | 318 | 4.66 | 35473.30 | nucleus |
| TkERF65 | CKM_transcript_15174 | 706 | 235 | 9.49 | 25971.49 | nucleus |
| TkERF66 | CKM_transcript_18427 | 664 | 221 | 9.91 | 24223.02 | nucleus |
| TkERF67 | CKM_transcript_19576 | 457 | 152 | 10.86 | 17052.19 | nucleus |
| TkERF68 | CKM_transcript_20186 | 499 | 166 | 6.60 | 18679.16 | chloroplast |
| TkERF69 | CKM_transcript_24855 | 955 | 318 | 4.66 | 35473.30 | nucleus |
| TkERF70 | CKM_transcript_25778 | 436 | 145 | 9.64 | 16591.61 | nucleus |
| TkERF71 | CKM_transcript_27223 | 595 | 198 | 7.87 | 21406.55 | nucleus |
| TkERF72 | CKM_transcript_27878 | 1168 | 389 | 5.02 | 43248.95 | nucleus |
| TkERF73 | CKM_transcript_28815 | 1168 | 389 | 5.02 | 43382.10 | nucleus |
| TkERF74 | CKM_transcript_29314 | 733 | 244 | 5.35 | 27145.29 | nucleus |
| TkERF75 | CKM_transcript_37790 | 1000 | 333 | 4.80 | 37372.44 | nucleus |
| TkERF76 | CKM_transcript_37955 | 1048 | 349 | 4.82 | 40256.19 | nucleus |
| TkERF77 | CKM_transcript_39049 | 255 | 84 | 5.26 | 8888.86 | nucleus |
| TkERF78 | CKM_transcript_39139 | 643 | 214 | 8.38 | 23609.60 | nucleus |
| TkERF79 | CKM_transcript_39917 | 919 | 306 | 4.83 | 34477.75 | nucleus |
| TkERF80 | CKM_transcript_52155 | 535 | 178 | 9.34 | 19642.21 | nucleus |
| TkERF81 | CKM_transcript_57358 | 709 | 236 | 9.53 | 26786.71 | nucleus |
| TkERF82 | CKM_transcript_58306 | 451 | 150 | 11.82 | 17458.29 | nucleus |
| TkERF83 | CKM_transcript_62381 | 583 | 194 | 8.78 | 21044.13 | nucleus |
| TkERF84 | CKM_transcript_62668 | 691 | 230 | 4.97 | 25092.80 | nucleus |
| TkERF85 | CKM_transcript_62988 | 763 | 254 | 7.60 | 28321.82 | nucleus |
| TkERF86 | CKM_transcript_64393 | 1168 | 389 | 4.92 | 43310.97 | nucleus |
| TkERF87 | CKM_transcript_69964 | 343 | 114 | 10.75 | 12989.02 | mitochondria |
| TkERF88 | CKM_transcript_71631 | 457 | 152 | 10.86 | 17075.23 | nucleus |
| TkERF89 | CKM_transcript_73539 | 598 | 199 | 9.89 | 22470.37 | cytoplasm |
| TkERF90 | CKM_transcript_74034 | 658 | 219 | 7.70 | 23889.41 | nucleus |
| TkERF91 | CKM_transcript_79271 | 1015 | 338 | 9.40 | 37190.13 | nucleus |
| TkERF92 | CKM_transcript_82772 | 574 | 191 | 8.72 | 21457.22 | nucleus |
| TkERF93 | CKM_transcript_83791 | 583 | 194 | 8.72 | 21597.37 | nucleus |
| TkERF94 | CKM_transcript_87379 | 763 | 254 | 6.77 | 28332.82 | nucleus |
| TkAP2-1 | CKF_transcript_7539 | 2112 | 703 | 6.60 | 76763.56 | nucleus |
| TkAP2-2 | CKF_transcript_8589 | 2104 | 701 | 6.57 | 76326.16 | nucleus |
| TkAP2-3 | CKF_transcript_11248 | 1480 | 493 | 6.68 | 53676.35 | nucleus |
| TkAP2-4 | CKF_transcript_11540 | 1435 | 478 | 7.69 | 52120.61 | nucleus |
| TkAP2-5 | CKF_transcript_13762 | 1339 | 446 | 5.76 | 49707.64 | nucleus |
| TkAP2-6 | CKF_transcript_16211 | 1294 | 431 | 8.88 | 47399.05 | nucleus |
| TkAP2-7 | CKF_transcript_16488 | 1507 | 502 | 6.11 | 55417.98 | nucleus |
| TkAP2-8 | CKF_transcript_18024 | 1369 | 456 | 8.99 | 50625.38 | nucleus |
| TkAP2-9 | CKF_transcript_28079 | 1882 | 627 | 6.87 | 69456.38 | nucleus |
| TkAP2-10 | CKF_transcript_36405 | 1357 | 452 | 10.13 | 50705.59 | nucleus |
| TkAP2-11 | CKF_transcript_37943 | 817 | 272 | 8.93 | 29965.09 | nucleus |
| TkAP2-12 | CKM_transcript_55971 | 820 | 273 | 8.90 | 29965.10 | nucleus |
| TkAP2-13 | CKF_transcript_58305 | 613 | 204 | 9.60 | 23170.99 | nucleus |
| TkAP2-14 | CKF_transcript_58669 | 1072 | 357 | 9.31 | 39496.10 | chloroplast |
| TkAP2-15 | CKF_transcript_62549 | 880 | 293 | 7.69 | 32217.74 | nucleus |
| TkAP2-16 | CKF_transcript_62749 | 1717 | 572 | 8.94 | 63427.74 | nucleus |
| TkAP2-17 | CKF_transcript_64620 | 898 | 299 | 9.40 | 33536.21 | nucleus |
| TkAP2-18 | CKF_transcript_66346 | 1135 | 378 | 6.34 | 41835.00 | nucleus |
| TkAP2-19 | CKF_transcript_68918 | 1441 | 480 | 6.90 | 52439.92 | nucleus |
| TkAP2-20 | CKF_transcript_71230 | 2101 | 700 | 6.58 | 76461.22 | nucleus |
| TkAP2-21 | CKF_transcript_88285 | 943 | 314 | 5.95 | 35507.29 | nucleus |
| TkAP2-22 | CKF_transcript_92689 | 1027 | 342 | 8.24 | 38502.38 | nucleus |
| TkAP2-23 | CKF_transcript_95485 | 1243 | 414 | 5.79 | 46669.18 | chloroplast |
| TkAP2-24 | CKF_transcript_100937 | 1435 | 478 | 7.22 | 51948.42 | nucleus |
| TkAP2-25 | CKM_transcript_14496 | 1738 | 579 | 8.06 | 64493.61 | nucleus |
| TkAP2-26 | CKM_transcript_23424 | 832 | 277 | 9.49 | 31278.10 | nucleus |
| TkAP2-27 | CKM_transcript_28644 | 1513 | 504 | 8.49 | 54138.11 | nucleus |
| TkAP2-28 | CKM_transcript_29021 | 421 | 140 | 10.31 | 16169.31 | nucleus |
| TkAP2-29 | CKM_transcript_40065 | 922 | 307 | 5.75 | 35419.47 | nucleus |
| TkAP2-30 | CKM_transcript_43459 | 1468 | 489 | 4.61 | 539953.22 | nucleus |
| TkAP2-31 | CKM_transcript_53049 | 796 | 265 | 8.42 | 29288.41 | nucleus |
| TkAP2-32 | CKM_transcript_62567 | 1738 | 579 | 8.28 | 64447.64 | nucleus |
| TkAP2-33 | CKM_transcript_67835 | 595 | 198 | 6.08 | 22887.49 | cytoplasm |
| TkAP2-34 | CKM_transcript_71764 | 1468 | 489 | 5.61 | 54007.31 | nucleus |
| TkAP2-35 | CKM_transcript_74774 | 799 | 266 | 5.63 | 29652.88 | nucleus |
| TkAP2-36 | CKM_transcript_81164 | 1321 | 440 | 5.78 | 49002.80 | nucleus |
| TkAP2-37 | CKM_transcript_83103 | 1108 | 369 | 5.46 | 41001.28 | nucleus |
| TkRAV1 | CKF_transcript_22278 | 988 | 329 | 9.47 | 36296.21 | nucleus |
| TkRAV2 | CKF_transcript_98416 | 1021 | 340 | 8.66 | 38801.86 | nucleus |
| TkRAV3 | CKM_transcript_65007 | 1021 | 340 | 8.51 | 38874.87 | nucleus |
| TkRAV4 | CKM_transcript_78648 | 1021 | 340 | 8.33 | 38818.84 | nucleus |
