# Supplementary figures and images for: Transcript-wide identification and expression pattern analysis to comprehend the roles of AP2/ERF genes under development and abiotic stress in Trichosanthes kirilowii
Source: BMC Plant Biol. 2023 Jul 10;23:354. doi: 10.1186/s12870-023-04362-0 (PMC10332102; doi:10.1186/s12870-023-04362-0)

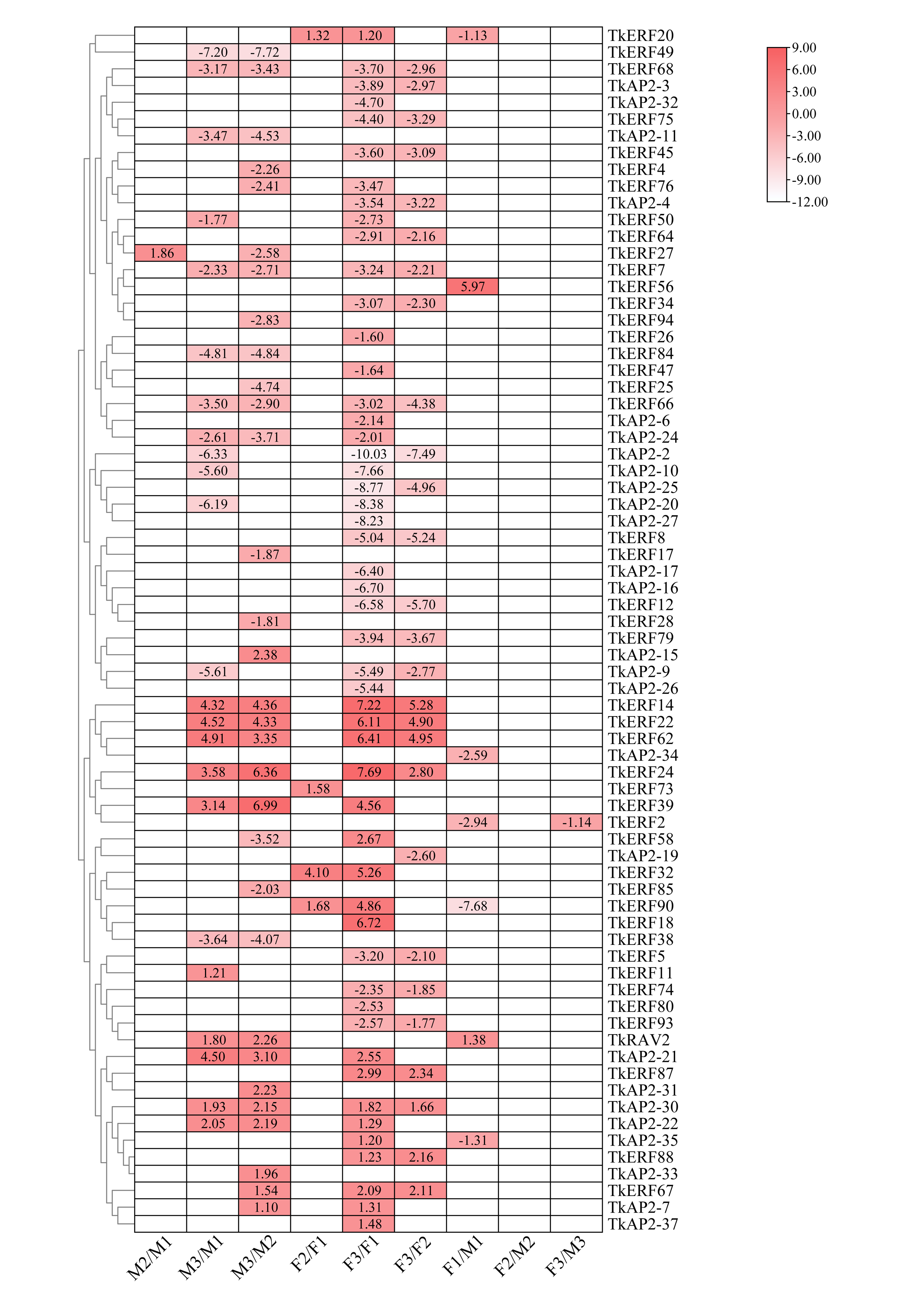

Supplement: Supplementary file 6 — Additional file 6: Figure S1. The expression trends of TkAP2/ERF gene family were analyzed based on the transcriptome data of female and male flowers at different flowering stages. M1, male buds; M2, male preliminary bloom; M3, male full-bloom; F1, female buds; F2, female preliminary bloom; MF3, female full-bloom. [file 12870_2023_4362_MOESM6_ESM.png]

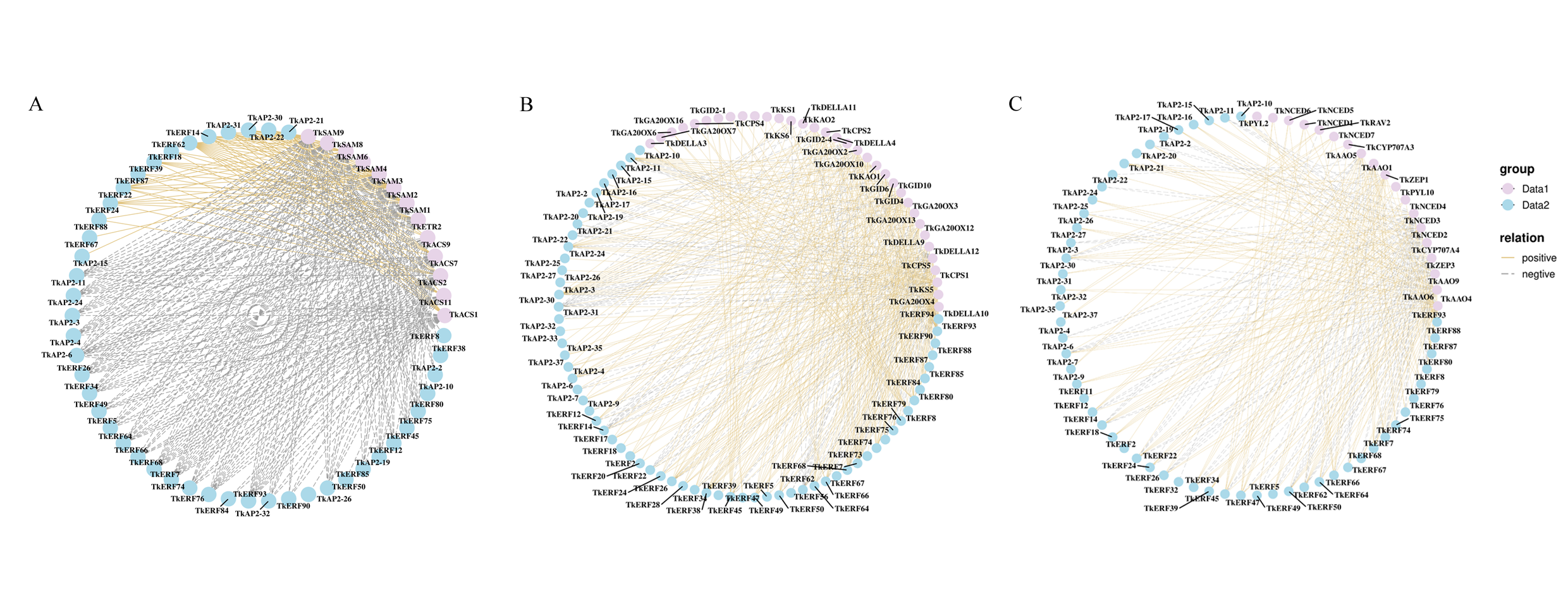

Supplement: Supplementary file 7 — Additional file 7: Figure S2. Co-expression networks based on transcriptome data of female and male flowers at different flowering stages. A: Co-expression network diagram of TkAP2/ERF differentially expressed gene and ethylene signaling pathway differentially expressed gene; B: Co-expression network diagram of TkAP2/ERF differentially expressed gene and gibberellin signaling pathway differentially expressed gene; C: Co-expression network diagram of TkAP2/ERF differentially expressed gene and abscisic acid signaling pathway differentially expressed gene. [file 12870_2023_4362_MOESM7_ESM.png]

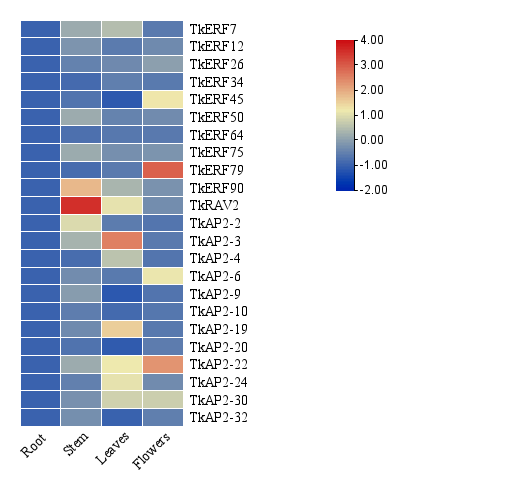

Supplement: Supplementary file 8 — Additional file 8: Figure S3. Expression profiles of Trichosanthes AP2/ERF genes across different tissues by qRT-PCR. [file 12870_2023_4362_MOESM8_ESM.png]
